# Analysis of cnidarian Gcm suggests a neuronal origin of glial EAAT1 function

**DOI:** 10.1038/s41598-023-42046-9

**Published:** 2023-09-08

**Authors:** Larisa Sheloukhova, Hiroshi Watanabe

**Affiliations:** Evolutionary Neurobiology Unit, Okinawa Institute of Science and Technology, 1919-1 Tancha, Onna-son, Kunigami-gun, Okinawa 904-0412 Japan

**Keywords:** Cell biology, Computational biology and bioinformatics, Evolution, Genetics, Molecular biology

## Abstract

In bilaterian central nervous systems, coordination of neurotransmission by glial cells enables highly sophisticated neural functions. The diversity of transcription factors (TFs) involved in gliogenesis suggests multiple evolutionary origins of various glial cell types of bilaterians. Many of these TFs including the *glial cells missing* (*Gcm*) are also present in genomes of *Cnidaria*, the closest outgroup to *Bilateria*, but their function remains to be elucidated. In this study, we analyzed the function of *Gcm*, a multifunctional TF involved in development of glial and non-glial cell types, in the sea anemone, *Nematostella vectensis.* siRNA-mediated knockdown of *Nematostella Gcm* altered expression of cell adhesion proteins, glutamate and GABA transporters, ion channels, metabolic enzymes, and zinc finger and *Ets*-related TFs. NvGcm and mRNAs of downstream genes are expressed in broad neural cell clusters. However, immunostaining of a NvGcm target protein, the glutamate transporter, NvEAAT1, visualized a novel class of cells with flat cell bodies and no clear processes. Together with the finding of unique morphological features of NvEAAT1-functioning cells, these data suggest that extracellular glutamate metabolism, one of major glial functions, is deployed downstream of *Gcm* in specific neural cell types in *Cnidaria*.

## Introduction

**Figure 1:**
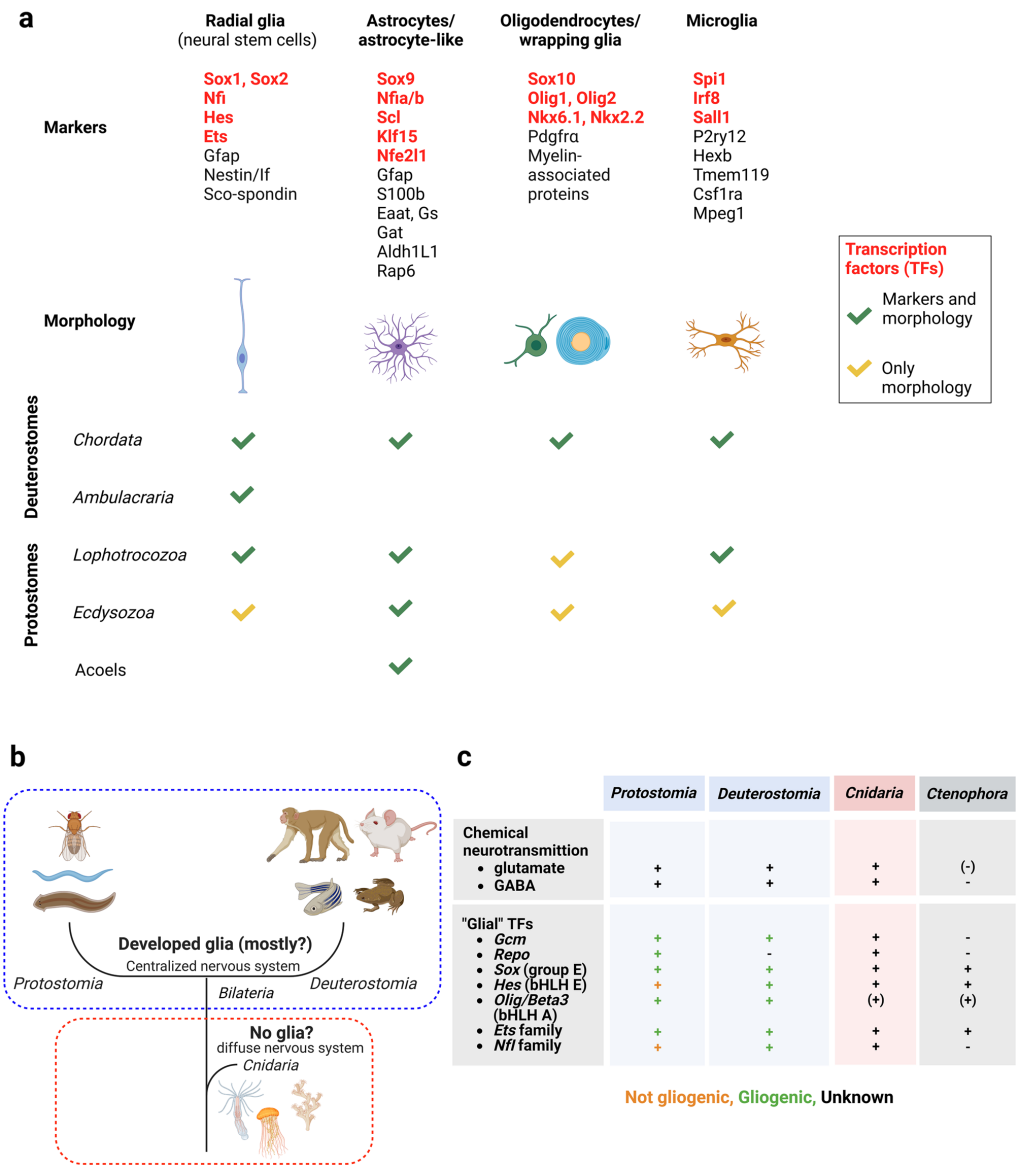
Prerequisites for glial origins in the Metazoa (**a**) Glial cell type conservation in bilaterian phyla. Molecular markers and morphological features of major glial cell types are shown. (**b**) The current view of glial evolution assumes that glia emerged with centralization of the nervous system, i.e., after the last common bilaterian ancestor diverged from the *Cnidaria.* (**c**) Nervous system features accompanying glial presence are conserved in *Cnidaria*, including all bilaterian gliogenic TFs.

Nervous systems (NS) of bilaterian animals are generally composed of neurons and glial cells. Neurons are the electrically active primary functional units of the NS. As support cells, glial cells participate in almost every process in bilaterian nervous systems**,** including neurotransmission, homeostasis, and development^1,2^. In the course of bilaterian evolution, glial cells have acquired greater morphological and functional diversity^2^, including distinct types, even within individual glial cell classes (Fig. 1a)^3^. Although the emergence of glial cells is one of the key novelties in evolution of the nervous system, extensive glial studies have been mostly confined to a few bilaterian model organisms, such as nematodes, fruit flies, zebrafish, rodents, and humans. Evolutionary origins of glia remain obscure.

Where did glial cells originate, and what were their first functions? Glial cells are hypothesized to have evolved with the appearance of a centralized nervous system, i.e., after the common bilaterian ancestor branched off from the *Cnidaria* (Fig. 1b)^2,4^. Gliogenic program conservation has not been investigated to date in *Cnidaria* or any other non-bilaterians.

*Cnidaria* and *Ctenophora* are the neuron-bearing phyla among non-bilaterians. Neurons of both phyla are organized in nerve nets with regional condensation sites and are neuropeptide-rich^5–8^. The *Cnidaria* is thought to share key features of the bilaterian NS, including a diverse repertoire of neurons and neurotransmitters, in addition to a complete set of bilaterian gene homologs driving neurogenesis and neuron specification^9–11^. Neuronal expression of glutamate and GABA/glycine vesicular transporters in *N. vectensis* suggests that chemical transmitters in secretory vesicles bearing these transporters are used as neurotransmitters in *Cnidaria* (Fig. 1c)^5^. Glutamate has been suggested as a candidate chemical messenger in *ctenophores*^12^. However, vesicular transporter expression is abundant in epithelial cells, and it is unclear whether glutamate functions as a neurotransmitter in this basal lineage^5^.

Incidentally, glutamate signaling modulation is one of the most prominent and conserved features of glial cells from *Caenorhabditis elegans*^13^ and *Drosophila melanogaster*^14^ to mammals^15^. Moreover, excitatory amino acid transporters (EAATs) are expressed in glia or glia-like cells of non-model invertebrates, including lancets^16^ and planarians^17^. To understand still enigmatic early evolution of glial system in neural functions, especially regulatory mechanisms of glutamate signaling, it is useful to analyze in cnidarians the function of gliogenic transcription factors (TFs) , endogenous drivers of glial cell identity in bilaterians. There are several TFs that drive generation of glial cells in the bilaterian nervous systems. In *Drosophila, Gcm* is a master regulator of gliogenesis. A mutation in this gene turns presumptive glia into neurons^18^. The glial program is activated by a downstream target of *Gcm, Repo,* which drives expression of glial-specific markers, including an *Ets* protein family member *Pnt* (pointed) (Fig. 1c)^19^. In vertebrates, gliogenetic mechanisms are more complicated, with multiple TFs, including not only *Gcm*, but also *bHLH*s, *Sox* group E, *Ets,* and *NFI* family proteins (Fig. 1c)^20–22^. Although vertebrate Gcm genes have diverse functions, *Gcm1*, a vertebrate *Gcm* ortholog, has neuro/gliogenic functions. For example, *Gcm1* drives neuronal differentiation in chickens^23,24^. Rodent *Gcm1* has the capacity to induce gliogenesis and to drive astrocyte differentiation^25,26^. The neural potential of *Gcm* homologs was also shown in other metazoans*.* For instance, *Gcm* expression confined to neurosecretory-like cells was demonstrated in freshwater crayfish^27^. Neuronal expression/function of Gcm homologues in various bilaterian lineages is of interest in regards to what the symplesiomorphy of Gcm function is.

In *Cnidaria*, a *Gcm* homolog is expressed in a subset of probable neuronal cells in *Nematostella vectensis*^7^, one of the most widely used cnidarian model organisms to address questions about evolution of the nervous system, thanks to the ease with which it can be cultured and to numerous established gene manipulation techniques^28,29^. Cell cluster enrichment analysis in a single-cell transcriptome of *N. vectensis* supports assumed neuronal functions of *NvGcm*^9–11^; however, the authors performed no functional exploration of its NvGcm-dependent gene regulatory network or characterization of *NvGcm*-expressing cells. This prompted us to investigate *NvGcm* and its candidate targets more carefully.

We performed *NvGcm* knockdown (KD) experiments using gene-specific siRNA and genome-wide screening of genes affected by Gcm depletion in *N. vectensis*. By characterizing transcripts affected by *NvGcm* KD, we identified genes involved in neurotransmission, in lipid and glucose metabolism, and cell adhesion molecules, suggesting a neural nature of the *NvGcm*-controlled program. Surprisingly, a previously undescribed cell type was revealed by immunostaining for the glutamate transporter EAAT1, a conserved target of *Gcm*. Morphologically these cells do not resemble any described *N. vectensis* neurons; therefore, they are a novel neural cell type. We use the term “neural” to denote cells including neurons and supporting cells like glial cells, whereas “neuronal” is used to denote neurons specifically. Therefore, we refer to *Nematostella* Eaat1-expressing cells as neural, but not necessarily neurons. These cells may represent “protoglia”, functioning primarily as glutamate-quenching cells for pepti-glutamatergic neurons that use both glutamate and neuropeptides as neurotrasnmitters.

## Results

Homologs of all bilaterian glial TFs, including *Gcm,* are found in a cnidarian model organism, *Nematostella vectensis*, but not in ctenophores (Fig. 1c). Moreover, among non-bilaterians, the *Gcm* domain is conserved only in the *Cnidaria* (Supplementary Fig. 1).

**Figure 2:**
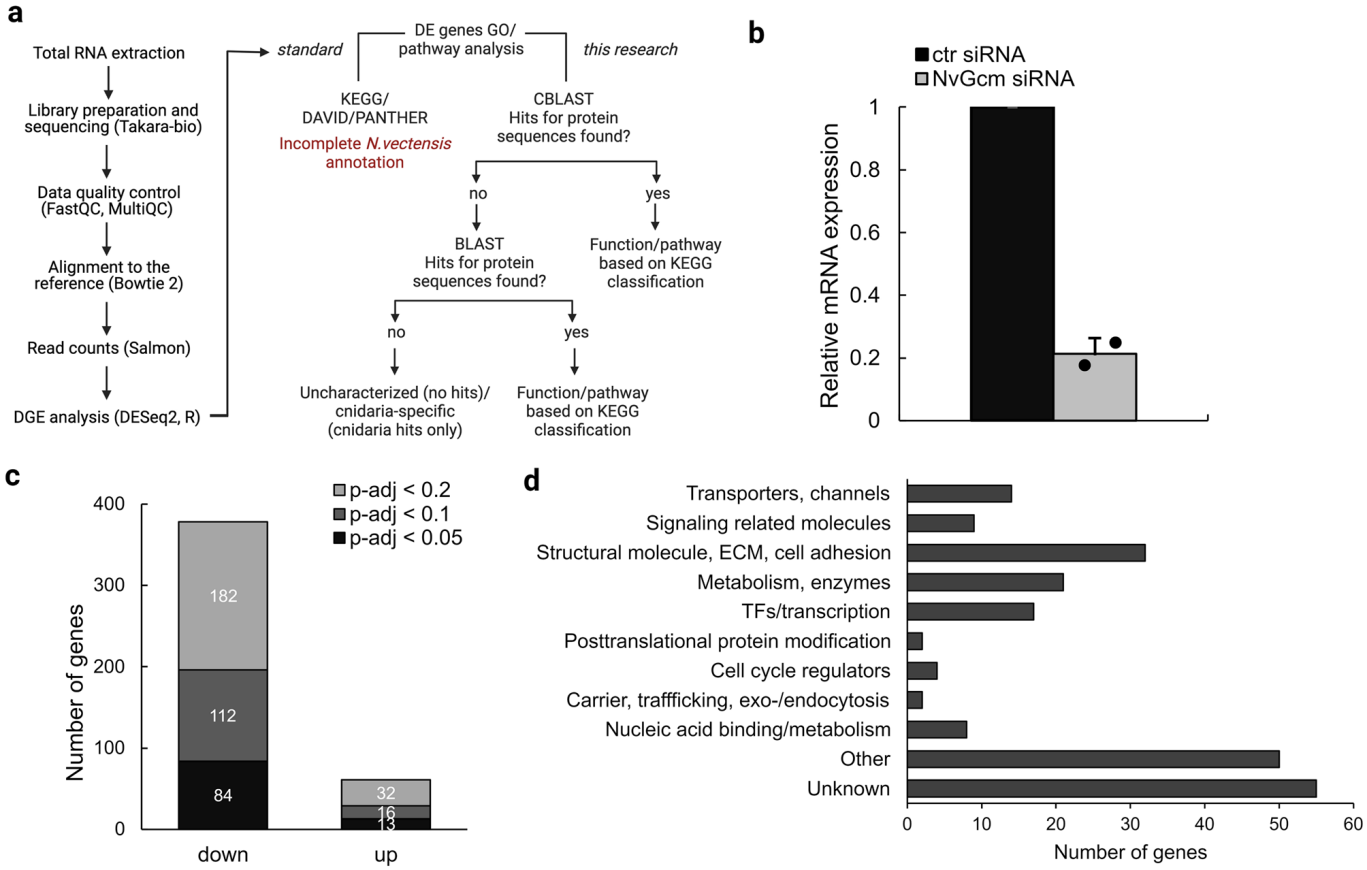
Transcriptomic analysis of *Gcm* KG in *N. vectensis*. (**a**) RNA-seq workflow used in this study (**b**) *NvGcm* KD efficiency of siRNA in experimental batches selected for RNA-seq analysis. (**c**) Total number of DE genes in *NvGcm*-depleted larvae depending on p-adj. (**d**) Functional classification of *NvGcm* candidate target genes.

To develop an unbiased view of *NvGcm* targets in *N. vectensis*, we knocked it down using *NvGcm*-specific siRNA^30^ and performed transcriptomic analysis (Fig. 2a). KD efficiency of *NvGcm*-targeted siRNA was ~ 75% for samples selected for RNA-seq analysis (Fig. 2b). To identify genes affected by *NvGcm* KD, we performed differential gene expression (DE) analysis and identified genes that were downregulated or upregulated in *NvGcm*-depleted larvae (Fig. 2c).

We next analyzed the composition of all DE genes (p-adj < 0.2, 214 genes) with respect to their pathway groups, as defined in the KEGG PATHWAY Database. All candidate genes were grouped into the same eleven functional categories to characterize *NvGcm* candidate target genes in *Drosophila* (Fig. 2d)^31^. Functions of about a fourth of the identified genes are unknown. As in *Drosophila*, transcription factors and genes encoding ion transporters and channels constitute 8% and 7% of affected genes respectively, suggesting a possible function in cell fate specification and homeostatic control of *NvGcm*. A large fraction (15%) of *NvGcm* candidate target genes are structural and cell adhesion molecules, whereas 4% are signaling-related molecules. Another 10% of these genes are enzymes involved in general metabolism. All DE genes and their functional classifications are listed in Supplementary Table 2.

**Figure 3:**
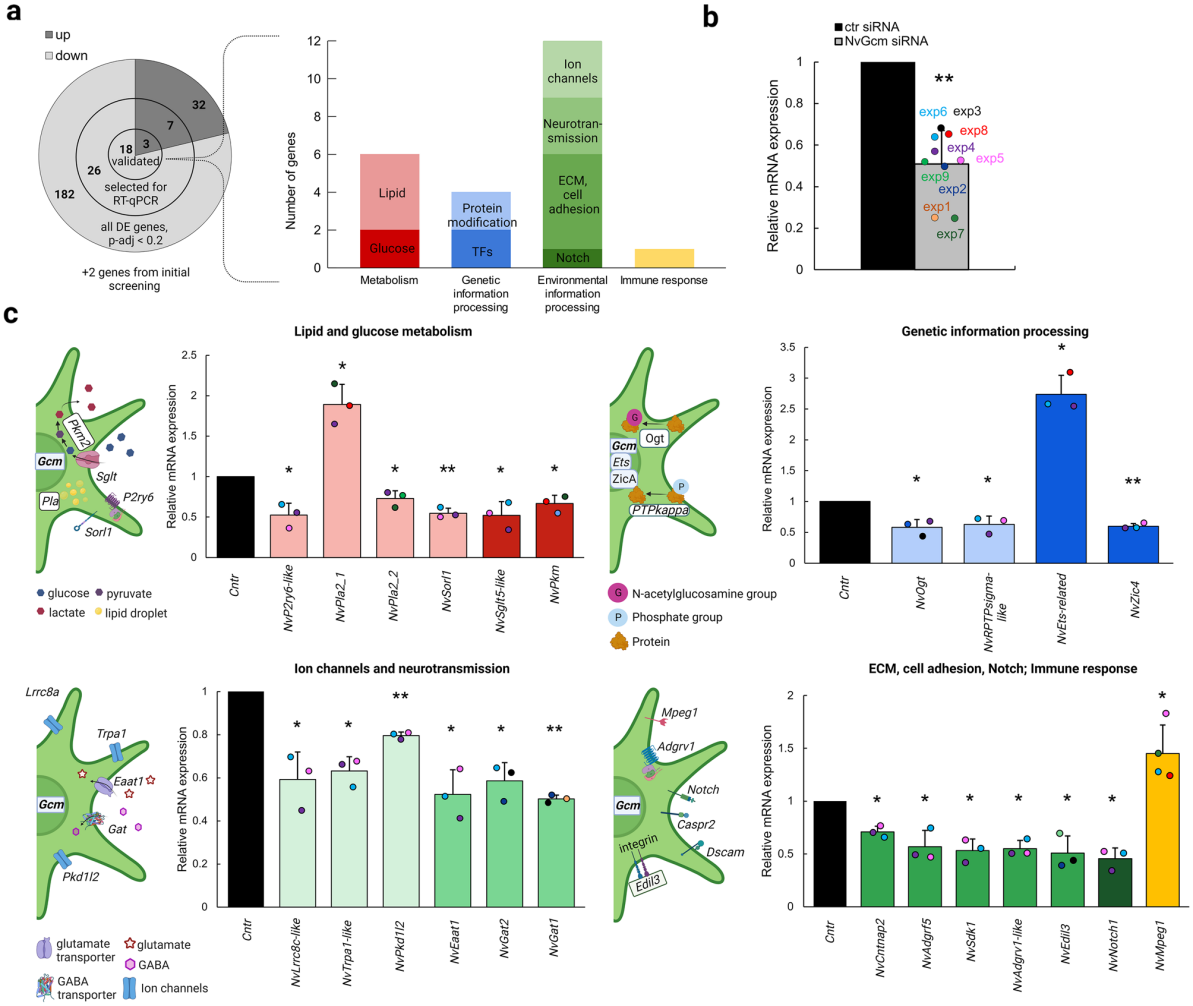
RT-qPCR validation of DE genes in *NvGcm*-depleted larvae. (**a**) Total number of DE genes identified in RNA-seq at p-adj < 0.2, numbers of DE genes selected for RT-qPCR validation, and numbers of genes validated by RT-qPCR. Functional classification of RT-qPCR validated DE genes (**b**) *NvGcm* KD efficiency in experimental batches used for RT-qPCR validation. Each experiment is color-coded. (**c**) Relative mRNA expression of validated candidate genes in *NvGcm*-depleted larvae compared to controls. Some up-regulated DE genes (*NvPla2.1*, *NvEts-related*, and *NvMpeg1*) are also shown. Each data point is color-coded and corresponds to the experiment in b. Simplified cartoons to the left of the bar graphs in c demonstrate cellular processes in which validated candidate genes are involved.

To further validate *NvGcm* candidate target genes, we selected several genes from each of the major GO groups for RT-qPCR analysis (Fig. 3a). We ignored those with functional groups that would not contribute to understanding the *NvGcm*-regulated program (unknown function, ncRNA/miscRNA, RNA splicing, ribosome, RT/retrotransposons, etc.). *NvGcm* KD efficiency was assessed with RT-qPCR for each experimental batch (Fig. 3b). We selected 33 genes for RT-qPCR validation and confirmed that 21 of them are up/downregulated in *NvGcm*-depleted larvae (Fig. 3a). In addition to these 21 genes identified in RNA-seq data, we confirmed that two genes encoding GABA transporters (*Gats*) are affected by *NvGcm* KD. We grouped validated *NvGcm* candidate target genes into nine functional categories, described below (Fig. 3a). All RT-qPCR validated candidate genes, their functional classifications, and RNA-seq attributes are listed in Supplementary Table 3.

Interestingly, NvGcm downstream targets in *Nematostella* included genes essential in bilaterian neuronal and glial functions. First, are genes encoding proteins involved in glucose and lipid metabolism: *Sorl1* (sortilin-related receptor 1), *Pla*_*2*_s (phospholipases A_2_), *P2Y6-like* (purinoceptor P2y6-like), *Sglt5-like* (sodium-dependent glucose cotransporter 5-like), and *Pkm (*pyruvate kinase) (Fig. 3c). Second, there are post-translational protein modification enzymes required for axonogenesis and axonal support: *RPTPsigma-like* (receptor-type tyrosine-protein phosphatase S-like) and *Og*t (UDP-N-acetylglucosamine–peptide N-acetylglucosaminyltransferase) (Fig. 3c)^32^. Third, there are TFs involved in development of the nervous system and in driving glio/neurogenesis: *Ets-related* and *Zic4* (zinc finger) (Fig. 3c)*.* Fourth, several ion channels and chemical neurotransmitter transporters are among the candidate genes: *Eaat1* (glutamate transporter), *Lrrc8a* (volume-regulated anion channel), *Gat*s (GABA transporters), *Pkd1l2* (transient receptor potential polycystic (*TRPP*) channel), and *Trpa1* (transient receptor potential cation channel) (Fig. 3c). Finally, candidate genes include cell adhesion and signaling molecules involved in organizing synapses and promoting glio-neuronal interactions: *Caspr2* (contactin-associated protein-like 2), *Adgrf5* (adhesion G protein-coupled receptor F5), *Sdk1* (sidekick 1), *ADGRV1-like* (adhesion G protein-coupled receptor V1-like), an integrin ligand *Edil3* (EGF-like repeat and discoidin I-like domain-containing protein, *Notch1,* and *Mpeg1* (Fig. 3c).

**Figure 4:**
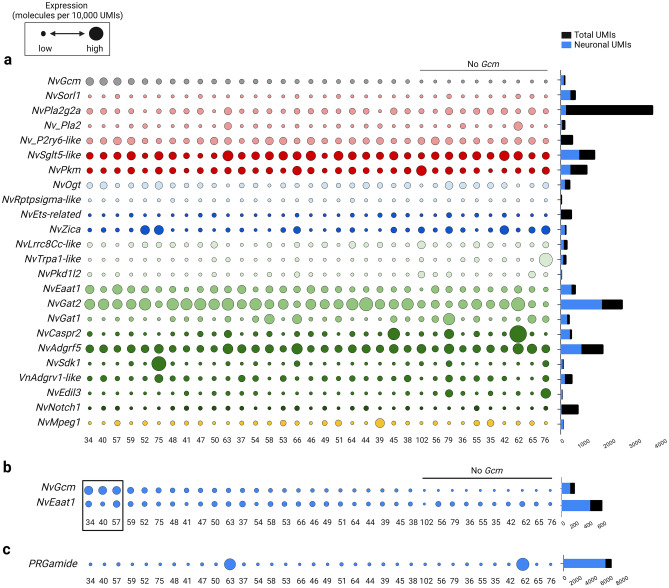
Genetic signature of *NvGcm*-expressing cells. Gene expression is based on scRNA-seq data (10). The dot plot shows normalized expression values (molecules per 10,000 UMIs) scaled by the gene. Smallest and largest dots represent the lowest and highest expression, respectively. Cell clusters are organized left to right in descending order of *NvGcm* expression. (**a**) Expression of *NvGcm* and its candidate target genes across neuronal cell clusters in adult *N. vectensis*. Dots are colored in accordance with gene functional groups as presented in Fig. 3. (**b**) Expression of *NvGcm* and *NvEaat1* across adult neuronal cell clusters. Cell clusters with the highest expression of *NvGcm* are framed (**c**) Expression of *PRGamide* neuropeptide gene across adult neuronal cell clusters.

We next analyzed expression profiles of NvGcm and its downstream genes using single-cell transcriptomic (SCT) data from adult *N. vectensis* to furnish an overview of functionally fully differentiated cell repertoires^10^. We found that *NvGcm* and most candidate genes are predominantly expressed in neuronal cell clusters (Fig. 4a, Supplementary Fig. 3a,b). *NvGcm* is broadly expressed across neuronal cell clusters, with the highest expression in C34, C40, and C57 (Fig. 4b, Supplementary Fig. 3c). Expression of *NvGcm* mRNA in a relatively broad range of cell types suggests several possible explanations. If it indicates the breadth of cell types in which this transcription factor actually functions, NvGcm may be involved in regulating expression of these downstream genes in various neurons, rather than deciding the fate of specific neuronal cell types, such as glia. Alternatively, alteration of gene expression by NvGcm depletion may be due, at least in part, to non-cell-autonomous effects. Among candidate genes, a glutamate transporter, *NvEaat1*, is of particular interest, as the highest expression of *NvEaat1* mRNA is found in C34 and C57, clusters enriched with *NvGcm* (Fig. 4b, Supplementary Fig. 3c). This is in sharp contrast to neuropeptides, e.g., PRGamide, that tends to be expressed only in specific neuronal clusters (Fig. 4c). In *Drosophila,* expression of excitatory amino acid transporters, *Eaat1* and *Eaat2*, depends on *Gcm*^33,34^, suggesting that *Eaat1* is a conserved *Gcm* target. Although these data reveal NvGCM-dependent gene expression, it is helpful to confirm protein expression to gain further insight into cells in which downstream genes function. Transcriptomes and proteomes tend to be correlated overall, but there are known exceptions^35,36^.

**Figure 5:**
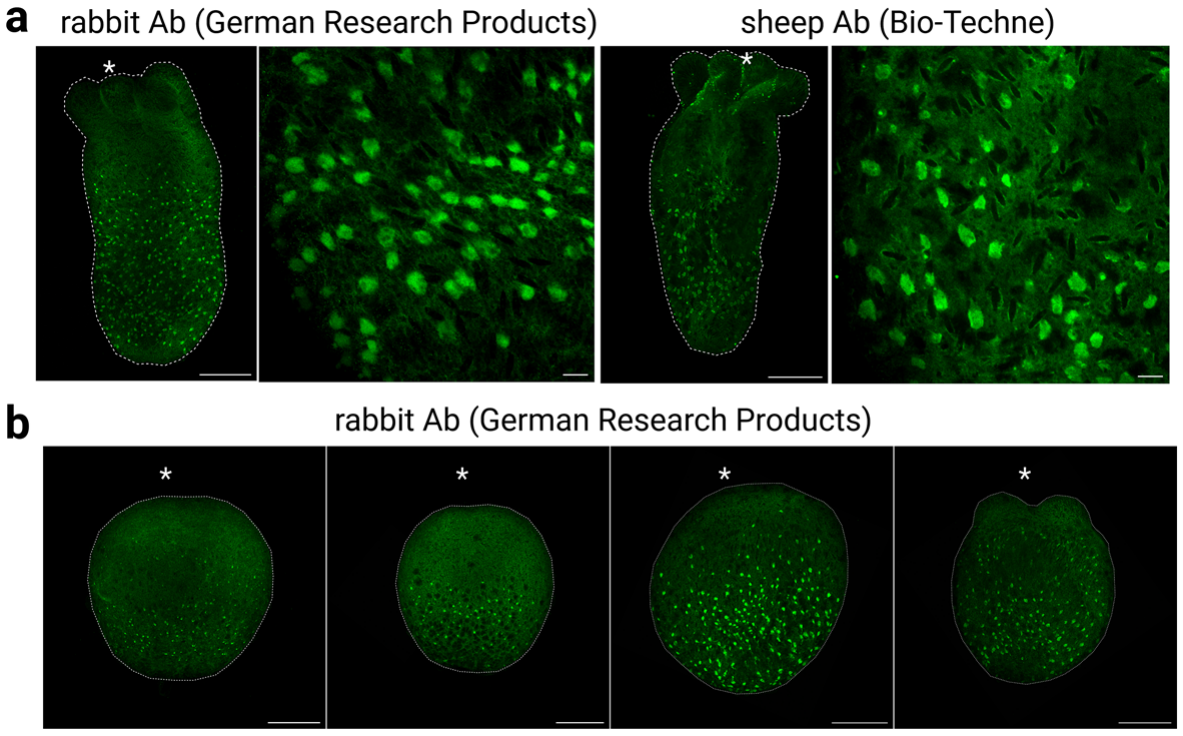
Morphology of NvEAAT1-positive cells. (**a**) EAAT1-expressing cells stained with German Research Products (rabbit) and Bio-Techne (sheep) anti-EAAT1 antibodies are broadly detected throughout the aboral region of the body column during the juvenile polyp stage (9dpf). Cells have flat morphology and lack processes. (**b**) Expression of NvEAAT1 at different developmental stages revealed by immunostaining with German Research Products anti-EAAT1 antibody. EAAT1-expressing cells became visible during larval stages. Side views of (from left to right): late gastrula stage (3dpf), early planula stage (4dpf), planula stage (5dpf), tentacle bud stage (6dpf). Asterisks indicate oral positions. Large and small scale bars are 100 μm and 10 μm, respectively.

To obtain information on morphological and physiological features of cells expressing glial genes, we first tried to identify cells expressing NvGCM protein using a specific antibody. However, our NvGCM antibody identified multiple bands in western blotting (Supplementary Fig. 4) and no clear signal in immunostaining, probably due to the low expression level of NvGcm mRNA (Fig. 4a). We next tried commercially available EAAT1 antibodies. The antibodies were selected based on the sequence similarity of the NvEAAT1 sequences and the epitopes declared to be recognized by the antibodies (Supplementary Fig. 5). An antibody from German Research Products gave a strong, specific signal in western blotting, while an antibody from Bio-Techne recognized multiple proteins of various molecular sizes (Supplementary Fig. 6a). Nevertheless, both EAAT1 antibodies detected a band with the expected size of NvEAAT1 (65 kDa) and identical staining patterns in immunostaining (Fig. 5a).

**Figure 6:**
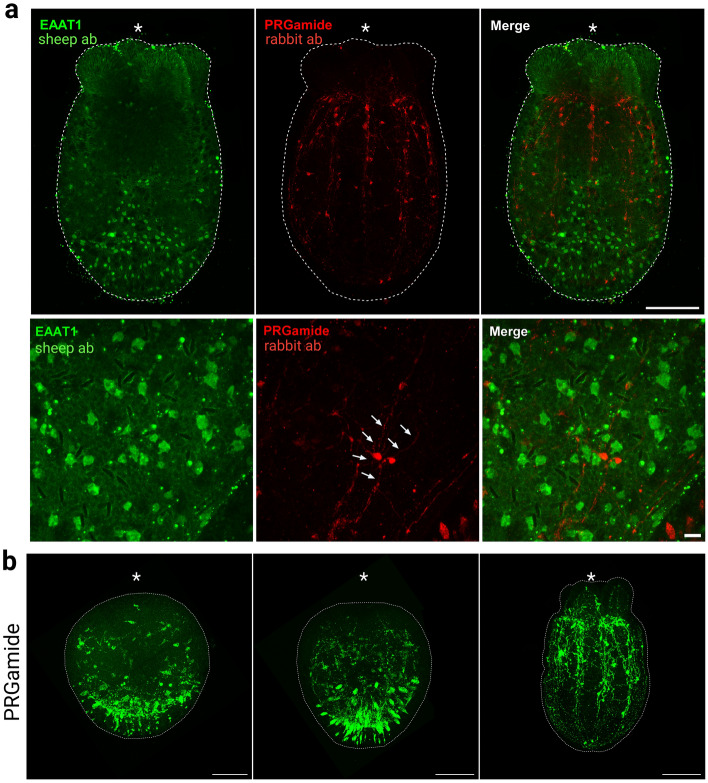
Non-overlapping expression of PRGamide and EAAT1 protein in the *N. vectensis* nervous system. (**a**) Immunofluorescence co-staining using anti-EAAT1 antibody (Bio-Techne) and anti-PRGamide antibody during the primary polyp stage (7dpf). EAAT1 staining and PRGamide staining do not overlap. EAAT1-positive cells lack processes, unlike PRGamide-positive neurons, which extend neurites (arrows). (**b**) PRGamide-expressing neurons detected at different developmental stages. Side views of (from left to right): early planula stage (4dpf), planula stage (5dpf), juvenile polyp stage (7dpf). Asterisks indicate oral positions. Large and small scale bars are 100 μm and 10 μm, respectively.

EAAT1-positive cells began to be detected at late gastrula stage in outer ectoderm of the aboral region (Fig. 5b). At late gastrula and early planula stages, only a few cells were detected. Numerous EAAT1-positive cells became visible in a broad aboral region from planula through primary polyp stages. The salt-and-pepper-like distribution of NvEAAT1-expressing cells in aboral epithelium is reminiscent of expression patterns of other genes expressed in neurons^37,38^. However, EAAT1-positive cells exhibited flat morphology without clear long neurites, a characteristic different from that of cnidarian interneurons with spherical or flask-shaped cell bodies (Fig. 6a, Supplementary Fig. 7). In fact, immunostaining for PRGamide, a neuropeptide most abundantly expressed in the NvGcm-enriched neuronal cluster during the larval stage (Supplementary Fig. 8), demonstrated typical morphology of neuropeptide-positive neurons at the aboral side of planula larvae (Fig. 6b).

Taken together, our functional analysis of NvGcm identified a number of Gcm downstream genes including neuronal/glial genes involved in glutamate quenching in *N. vectensis*. Although mRNAs of NvGcm and many of its downstream genes, including EAAT1, were widely expressed in various neuronal clusters, EAAT1 protein was visualized mainly in a population of cells with non-neuronal morphology.

## Discussion

The glial cell system is highly developed in the central nervous system of various bilaterian lineages. This suggests that glial cell function is required for sophisticated nervous systems. To understand evolution of the neuro-glial system, it is necessary to understand how glial cells became functionally deployed in early stages of evolution. However, glial function in the nervous system in pre-bilaterians is still unknown. In this work, for the first time, we analyzed Gcm function in non-bilaterian animals.

Although it is not clear what functions NvGcm-target genes have, they can be assumed from what is known about bilaterian homologs of these genes. NvGcm-dependent genes include a TF homolog of the zinc finger protein family, *Zic4*, which is required for development of the nervous system in mice, frogs, and humans^39,40^, as well as homologs of post-translational protein modification enzymes, *RPTPsigma* and *Ogt. RPTPsigma* is indispensable for axon guidance and axonogenesis in bilaterians^32^. *Ogt* is an important metabolic sensor involved in glycolysis regulation, which mediates glia-neuron interaction by promoting glial axonal support^41^. Surprisingly, we found that expression of an *Ets* domain-containing TF increased in *NvGcm*-deficient larvae. Conversely, in *Drosophila*, *Gcm* induces expression of a glia-specific *Ets* protein family member, *Pnt* (pointed domain) via a homeobox TF, *Repo*. *Ets* family genes are a large group of TFs, some of which also drive gliogenesis in vertebrates^42–44^. In *N. vectensis*, 12 *Ets* genes were found, with *NvGcm* being a member of an apical pole gene regulatory network of a *Pointed*-containing *Ets* gene, *NvErg*^45,46^. An *Ets* gene we identified as a *NvGcm* candidate target in this study, does not contain a pointed domain and is not an obvious homolog of a specific bilaterian *Ets* protein. Its function in *N. vectensis* is unknown.

Future studies in *N. vectensis* should clarify the extent to which the *Gcm-Ets* axis is functionally conserved in non-bilaterians. In *Drosophila, Repo* is an important downstream target of *Gcm*, driving gliogenesis. However, in *N. vectensis* expression patterns of *NvRepo* and *NvGcm* do not overlap^7^, and our RNA-seq data did not contain *NvRepo* TF as a DE gene in *NvGcm*-deficient larvae. Moreover, the two TFs are most abundant in different cell clusters at both adult and larval stages (Supplementary Fig. 9). In adult animals, the neuronal cell cluster with the highest expression of *NvRepo* is devoid of *NvGcm*. Similarly, during the larval stage, *NvRepo* is most abundant in *NvGcm-*negative cell clusters. At both adult and larval stages, gland/secretory cell clusters demonstrate the highest expression of *NvRepo,* unlike *NvGcm*, indicating a non-neuronal function of *Repo*. Given that *Repo i*s not reported to have a gliogenic role in animals other than insects, with vertebrates lacking the gene altogether, the *Gcm-Repo* axis may have been established during arthropod evolution.

As with *Drosophila Gcm, NvGcm* candidate target genes include genes with known metabolic functions in bilaterians. These include sodium/glucose co-transporter, *NvSglt5-like*, and a pyruvate kinase, *NvPkm*, mediating the last step of glucose conversion to pyruvate, as well as receptors, *NvSorl1* and *NvP2ry6*, and enzymes, *NvPlas,* involved in lipid metabolism. This suggests that *N. vectensis Gcm*-expressing cells are metabolically active in ways resembling astrocytes and *Drosophila* glia, which store energy in the form of glycogen and lipid droplets for subsequent nutrient supply to neurons^47–50^. On the other hand, an increased expression of inflammatory marker homologs in *NvGcm* KD animals, a phospholypase *PLA*_*2*_^51^ and an immune effector, *Mpeg1*^52,53^, may indicate unknown involvement in the anti-inflammatory function of *NvGcm*. This is consistent with the recent finding of a conserved anti-inflammatory transcriptional cascade of *Gcm* from *Drosophila* to vertebrates^54^.

We identified homologs of several cell adhesion molecules, G-protein-coupled receptor and signaling proteins as *NvGcm* candidate targets abundant in neuronal cell clusters (Fig. 4a). This suggests that *NvGcm*-expressing cells actively participate in organizing the nervous system during *N. vectensis* development. Reduced expression of ion channels and neurotransmitter transporters in *NvGcm*-depleted larvae indicates functional involvement of these cells in chemical neurotransmission. Although in our RNA-seq data no plasmalemmal GABA transporters were affected by *NvGcm* KD, we analyzed expression of three *NvGats* in *NvGcm*-depleted larvae and found a reduction of two *NvGats* (Fig. 3c). This suggests possible involvement of *Gcm*-expressing cells in GABA uptake following neurotransmission in *N. vectensis*. However, it is still debated whether GABA is used as a neurotransmitter in cnidarians^5,11^. For instance, most expression of glutamic acid decarboxylase (GAD), the enzyme that catalyzes conversion of glutamate to GABA, is confined to non-neural cells, such as gastrodermis. Glutamate, on the other hand, is likely released via vesicular packaging from neurons, based on the gene expression profile of glutamate neurotransmission machinery in *N. vectensis*^5^*.* It is interesting to note that the emergence of the Gcm gene coincides with the appearance of neurons that use glutamate as a chemical transmitter.

Immunostaining of NvEAAT1 revealed a previously undescribed cell type, scattered throughout the aboral ectoderm during both larval and polyp stages of *N. vectensis* (Fig. 5). Although cell clusters with abundant expression of *NvGcm/NvEaat1* in the adult SCT are classified as neuronal because of their enrichment with classical neuronal markers, in at least some of those cells, the function of quenching extracellular glutamate is under control of *Gcm*.

Due to the lack of successful visualization of glutamate-releasing neurons, further investigation is needed to determine how cells expressing EAAT1 protein are spatially and physiologically associated with glutamatergic neurons. The extent of functional diversity of neurons in the *Cnidaria* is still unclear. However, the diversity of genetic signatures exhibited by neuronal clusters of *N. vectensis* at the mRNA level, taken together with the possible presence of additional regulatory mechanisms at the protein translation level, implies potential richness of neuronal functional variation. For example, we currently do not know the reason for the discrepancy between the broad expression profile of NvEAAT1 mRNA seen on dot plots and the restricted expression pattern in immunostaining of NvEAAT1 protein. Perhaps NvEAAT1 protein expression is also regulated at the translational as well as transcriptional level. In addition, it is not yet clear whether the cells visualized by the EAAT1 antibodies are a type of neural cell. However, the fact that the *Gcm* and Eaat1 genes are predominantly expressed in neuronal clusters in cnidarians suggests that cell types with developed glial functions have an evolutionary origin as “protoglia”, which had both neuronal and glial features. It is assumed that a new cell type emerges by a modification of a pre-existing cell type^55,56^. It is possible that the last common ancestor of *Bilateria* and *Cnidaria* possessed cells combining characteristics of both neurons and glia, which were inherited by both animal groups and subsequently diverged. Later through the “division of labor” various glial cell types emerged in the *Bilateria.*

## Methods

### Phylogenetic analysis of GCM

Bidirectional BLAST^57,58^ searches using the *Drosophila melanogaster* GCM1 protein sequence were performed using databases of representative bilaterians and non-bilaterians (Supplementary Table 1). Fungal protein sequences with the highest similarity to animal GCM were used as an outgroup. Sequences were aligned using MUSCLE (Mega7) and trimmed by eye to include only the domain. Trees were constructed with the maximum likelihood (ML) method using PhyML (SeaView). Bootstrap support is based on 2000 replicates.

### Nematostella vectensis culture

*N. vectensis* was cultured as described previously^28^.

### RNA interference

For *NvGcm* and *NvEaat1* RNA interference by transfection, *N. vectensis* embryos were electroporated as described in the previous report^59^ with the following modifications. No Ficoll was added to the brackish water medium because it decreased embryo survival. siRNA was used instead of shRNA due to the simplicity of siRNA manufacturing. Egg masses were collected, de-gelled, and fertilized prior to transfection. Electroporation was carried out using a Gene Pulser Xcell electroporation system (BIO-RAD).

*NvGcm*-specific and *NvEaat1*-specific siRNAs sequences were designed to knock down those genes (Supplementary Table 4). Updated *N. vectensis* genome assembly (NCBI, 20 Mar 2021) distinguishes two *Gcm* genes: 5,495,825 and 5,504,408 (IDs). Because of the high similarity of the sequences (98%), it is technically difficult to separate the two genes and to analyze their protein functions independently. The two *NvGcm*s are not distinguished in single-cell data^10^. In our study we do not separate them either. Instead, *NvGcm*-targeted siRNAs and *NvGcm* primers recognize both paralogs. Control embryos were electroporated with a negative control siRNA that was not complementary to any part of the genome (Supplementary Table 4). An siRNA concentration of 500 ng/uL was used. 4dpf *N. vectensis* planulae were collected for RNA extraction and knock-down efficiency assessment employed RT-qPCR.

### RNA extraction

Total RNA was extracted from *N. vectensis* planula larvae 4 days post-fertilization (dpf) using an RNeasy Mini Kit per manufacturer's guidelines (QIAGEN). Total RNA was dissolved in 30 μL RNase-free water and used immediately for, or stored at − 80 °C prior to cDNA synthesis. A NanoDropTM 1000 Spectrophotometer (Thermo Fisher Scientific) was used to check RNA concentrations and purity. Samples with absorbance ratios OD260/OD280 and OD260/OD230 higher than 1.6 were used. Extracted RNA for RNA-seq experiments was quality-checked using an Agilent TapeStation (Takara Bio facility). RIN (RNA integrity number) values of all samples were confirmed to be 10, i.e., sufficient for high-quality sequencing.

### Next-generation sequencing

Total RNA was extracted from five biological replicates in each of the three groups: *Gcm* KD siRNA1, *Gcm* KD siRNA2, and control siRNA. RNA extracted from all 15 samples was placed on dry ice and sent to Takara for sequencing. Library preparation (TruSeq RNA Library Prep Kit) and sequencing (NovaSeq6000) were also done by Takara bio (paired-end: 150 bp × 2; depth: 50–80 ml). *Gcm* KD efficiency in RNA samples used for sequencing was assessed with RT-qPCR analysis. RNA from several knock-down experiments was used to confirm DE genes identified by RNA-seq data analysis.

### RNA-seq data quality control and read mapping

Read quality was assessed using FastQC (Andrews, 2010) and visualized with the MultiQC python package^60^. These data were of good quality and no trimming was required. Paired reads were aligned to the most updated *N. vectensis* gene models available on Figshare using Bowtie 2 aligner^61^ with prior filtering using CD-HIT-EST (similarity threshold set to 94%) to remove isoforms^62^. The overall mean alignment rate was 76% (Supplementary Table S5). SAMtools was used to convert files from SAM to BAM format. Salmon^63^ was used to quantify transcript abundances. Salmon-generated pseudo counts, represented as normalized TPM (transcripts per million), were converted into non-normalized count estimates for differential gene expression analysis. RNA-seq profiles of 23,913 transcripts were obtained.

### Differential gene expression analysis

To identify genes differentially expressed (DE) in *Gcm* KD and control groups, Bioconductor DESeq2 was used^64^. Sample-level quality control (QC) was performed using Principal Component Analysis (PCA) and hierarchical clustering methods. Two of five experimental batches were selected to ensure that experimental conditions constituted the primary source of variation. *Gcm* KD for both siRNA1 and siRNA2 was most efficient in these batches (Supplementary Fig. 10). Experiment dates represented another source of variation, which was controlled during DEseq analysis. *Gcm* KD siRNA1 and siRNA2 groups were compared to the control group separately. The P-adj (false discovery rate) was set to 0.05, 01, and 0.2. Results for siRNA2 vs. control are shown because this siRNA was consistently more efficient and yielded more DE genes. DE genes for siRNA1 vs control are presented in Supplementary Table 6.

### Functional analysis of DE genes

Available databases used for gene ontology (GO)/pathway analysis such as KEGG, PANTHER, and DAVID have incomplete *N. vectensis* annotations. Therefore, in order to categorize DE genes into functional groups and explore their molecular pathways, each protein function was inferred from its similarity to bilaterian proteins using CBLAST (protein data bank)/BLAST^57,58^. Genes were then assigned to pathway groups as defined in the KEGG PATHWAY Database^65^.

### Real-time qPCR

Single-stranded cDNA was synthesized from 300 to 500 ng of total RNA in a final volume of 20 µL using oligo(dT)20 and SuperScript IV Reverse Transcriptase (Invitrogen), according to manufacturer’s instructions. cDNA was stored at − 20 °C for future use. Agarose gels of PCR products obtained using primers for a gene not affected by Gcm KD as confirmed by RT-qPCR were run to check for genomic DNA contamination of cDNA. Primers were designed to yield PCR products of different lengths for cDNA and gDNA (Supplementary Table 7). RT-qPCR was performed using a StepOne Plus™ Real-Time PCR System (Thermo Fisher Scientific). The amplification program was set as follows (fast mode ~ 40 min): holding stage at 95 °C for 20 s followed by 40 cycles of 3 s at 95 °C and 30 s at 60 °C. After amplification, a denaturing cycle (15 s at 95° followed by 1 min at 60 °C and 15 s at 95 °C) was performed to obtain melting curves and to verify amplification specificity. Samples for RT-qPCR were prepared using PowerUp SYBR® Green Master Mix, as per the manufacturer’s instructions. Primers were designed using Geneious 10.2.4 and Primer-BLAST^66^. Primer sequences and product sizes are listed in Supplementary Table 7. Primer efficiencies were assessed using cDNA fivefold serial dilutions through generation of a standard curve. Primers with 80–110% efficiency were chosen for qPCR analysis. Four samples were run for each gene. Triplicates were used for analysis. In qPCR data normalization, Norm-Finder^67^ was used to assess variability of three candidate housekeeping genes, *Gapdh*, *Ef1a*, and *18S*, and how much they are affected by experimental conditions, which are important factors to consider when choosing normalization genes^68^. *Ef1a* was consistently the best candidate among the three with the lowest intra- and intergroup variation (3 experiments, 4–8 samples per group, 3 groups: siRNA1, siRNA2, control siRNA). As a result*,* expression of genes of interest was normalized to *Ef1a*. The comparative Ct (△△Ct) value method was used for relative quantification^69^. RT-qPCR data are presented as expression fold change (± SD) normalized to one endogenous control (*Ef1a*) and relative to control siRNA. Means of different groups were compared (siRNA2 and siRNA control) and analyzed using Student’s unpaired t-test. Results from three independent experiments were used for the analysis unless stated otherwise. Differences were considered statistically significant when the *p*-value was < 0.05.

### Immunostaining and western blotting

To detect NvEAAT1 two polyclonal antibodies were used: rabbit anti-EAAT1 antibody (German Research Products, 1:400 for IF and 1:1000 for WB), sheep anti-EAAT1/GLAST-1 antibody (Bio-Techne, Cat. #AF6048, 10 μg/mL for IF and 5 μg/mL for WB). To detect PRGamide, an affinity-purified rabbit IgG raised against amidated Cys-PRGamide (1:300 for IF)^5^. The following secondary antibodies were used: Alexa Fluor 488-conjugated anti-rabbit and anti-sheep antibodies (goat, Jackson ImmunoResearch, 1:500) for IF; Alexa Fluor 647-conjugated anti-rabbit antibodies (goat, Jackson ImmunoResearch, 1:500) for IF; anti-rabbit and anti-sheep peroxidase-conjugated antibodies (goat, Jackson ImmunoResearch, 1:10,000) for WB. For indirect immunostaining, bud/polyp stage *Nematostella* were anesthetized using 2.43% MgCl_2_ for 10 min. Planulae and anesthetized bud/polyps were fixed in ice-cold 4% PFA/PBS + 0.1% Triton X-100 (PBSTx) for 1 h at RT on a rotator. Samples were washed 3 times (10 min each) with PBS + 0.1%Tween (PBT). Samples were then incubated in blocking solution (PBT/1%BSA/5% Normal goat serum/0.01%NaN3) for 1 h at RT, followed by o/n incubation with primary antibodies at 4 °C on the rotator. For double immunostaining, samples were incubated with sheep anti-EAAT1 antibody (Bio-Techne) and rabbit anti-PRGamide antibody simultaneously. Samples were then washed 2 times (10 min each) with PBSTx and incubated in the blocking solution for 1 h at RT on the rotator. Samples were incubated in secondary antibodies for 1 h at RT on the rotator. Samples were then washed 3 times with PBSTx (15 min each) at RT. Samples were then incubated for 30 min in DAPI (1:1000) and mounted with SlowFade Gold Antifade Mountant (Invitrogen). Western blotting was carried out as described elsewhere^70^.

### Microscopy

Images were captured on a Zeiss LSM780 confocal microscope system using × 40 and × 63 objectives. Image manipulation was performed with ZEISS ZEN microscope software and subsequently with ImageJ.

### Supplementary Information

Supplementary Information.

## Data Availability

RNA sequencing results were deposited in DNA Data Bank of Japan Sequence Read Archive under submission number DRA016214. Code used for RNA-seq analysis is available upon request.
