# Supplementary material for: Analysis of cnidarian Gcm suggests a neuronal origin of glial EAAT1 function
Source: Sci Rep. 2023 Sep 8;13:14790. doi: 10.1038/s41598-023-42046-9 (PMC10491807; doi:10.1038/s41598-023-42046-9)
Supplement: Supplementary file 1 — Supplementary Information. [file 41598_2023_42046_MOESM1_ESM.pdf]

Analysis of cnidarian Gcm suggests a neuronal origin of glial EAAT1 function.

Larisa Sheloukhova and Hiroshi Watanabe*

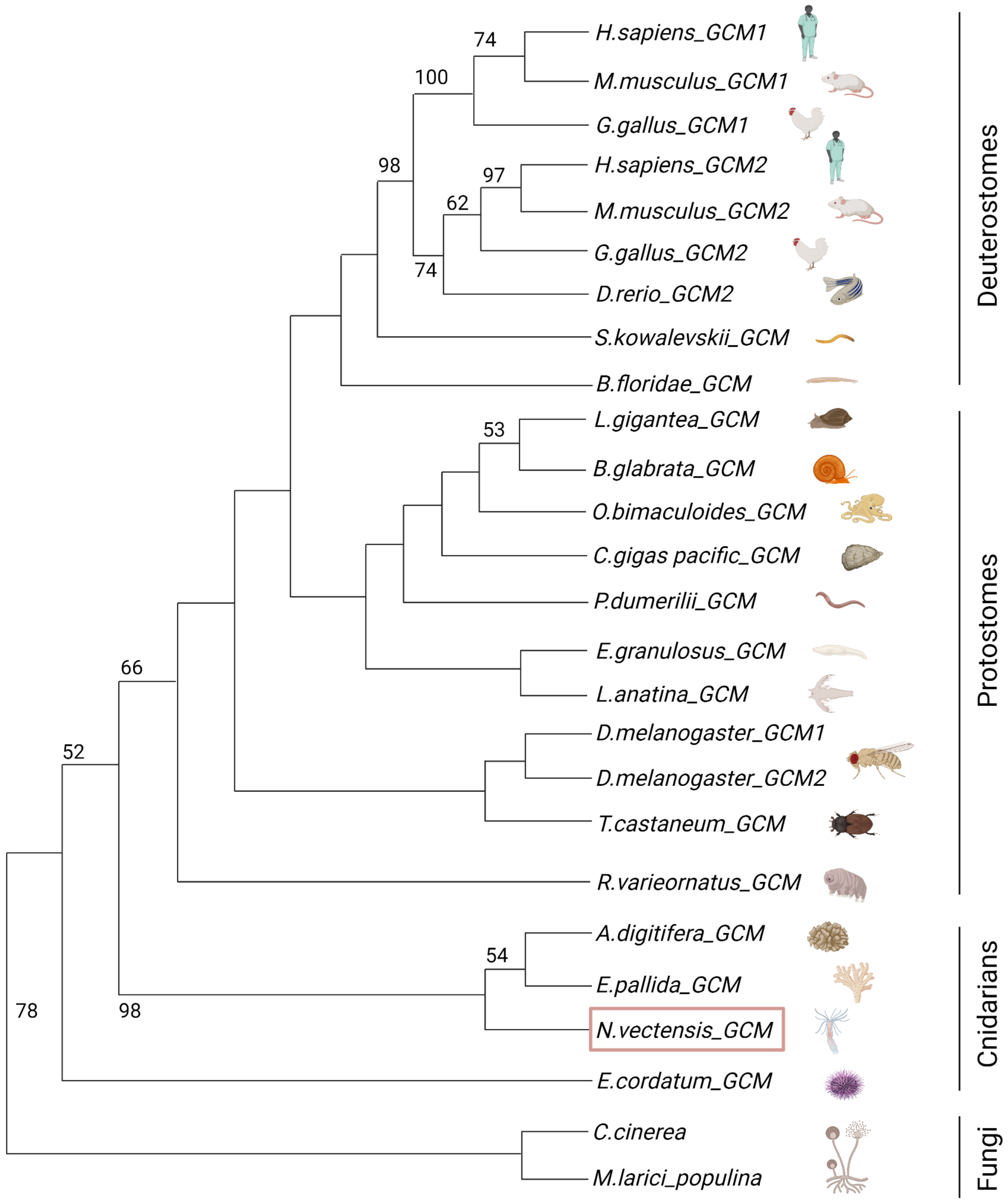

Supplementary Figure 1. Phylogeny of GCM in metazoans. Among basal metazoans, the GCM domain is conserved only in *Cnidaria*. Support values >50 are shown at the basal nodes. *Nematostella* GCM is framed in red.

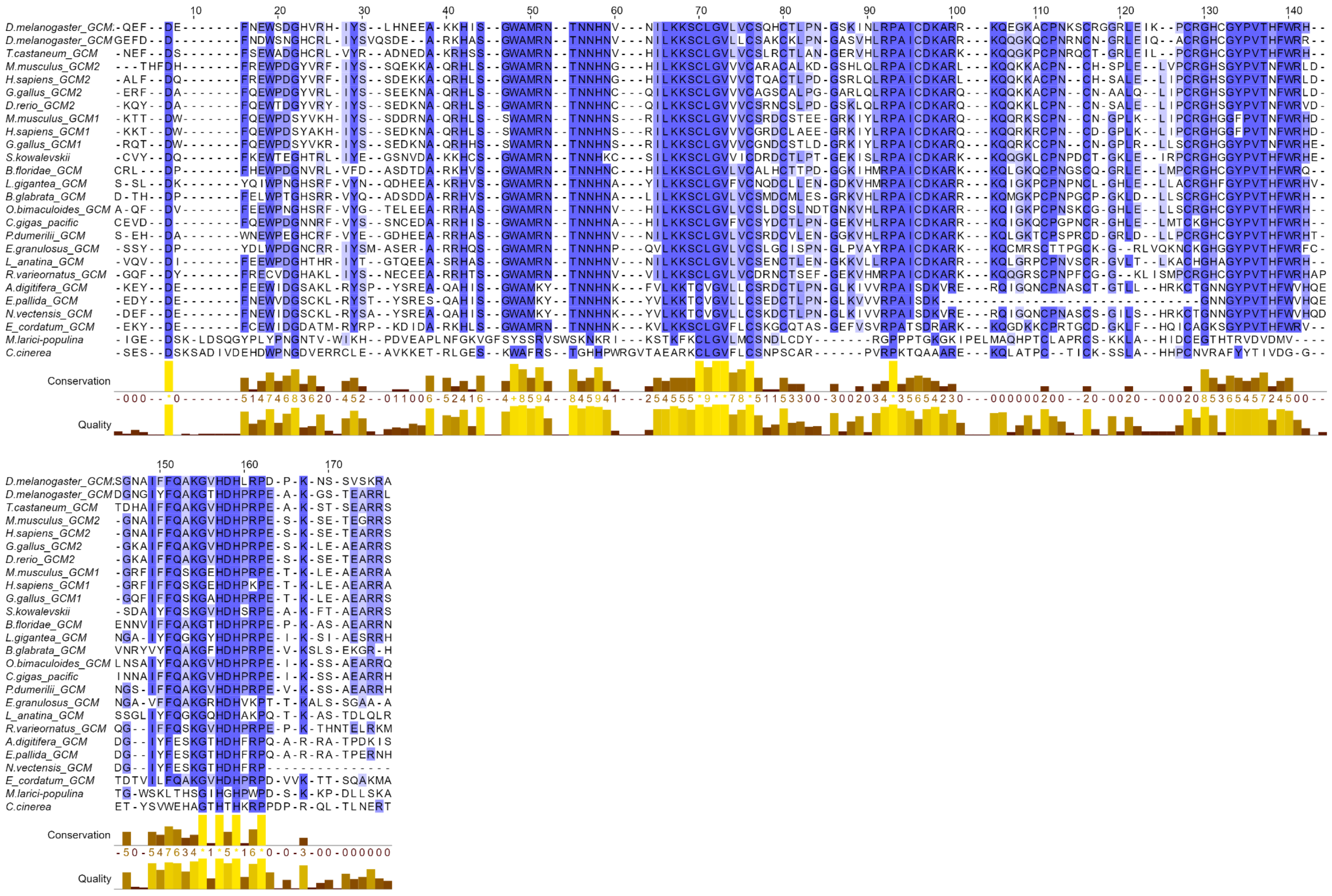

Supplementary Figure 2. Alignment of GCM domain. Created with JalView Version 2 (Waterhouse et al., 2009)

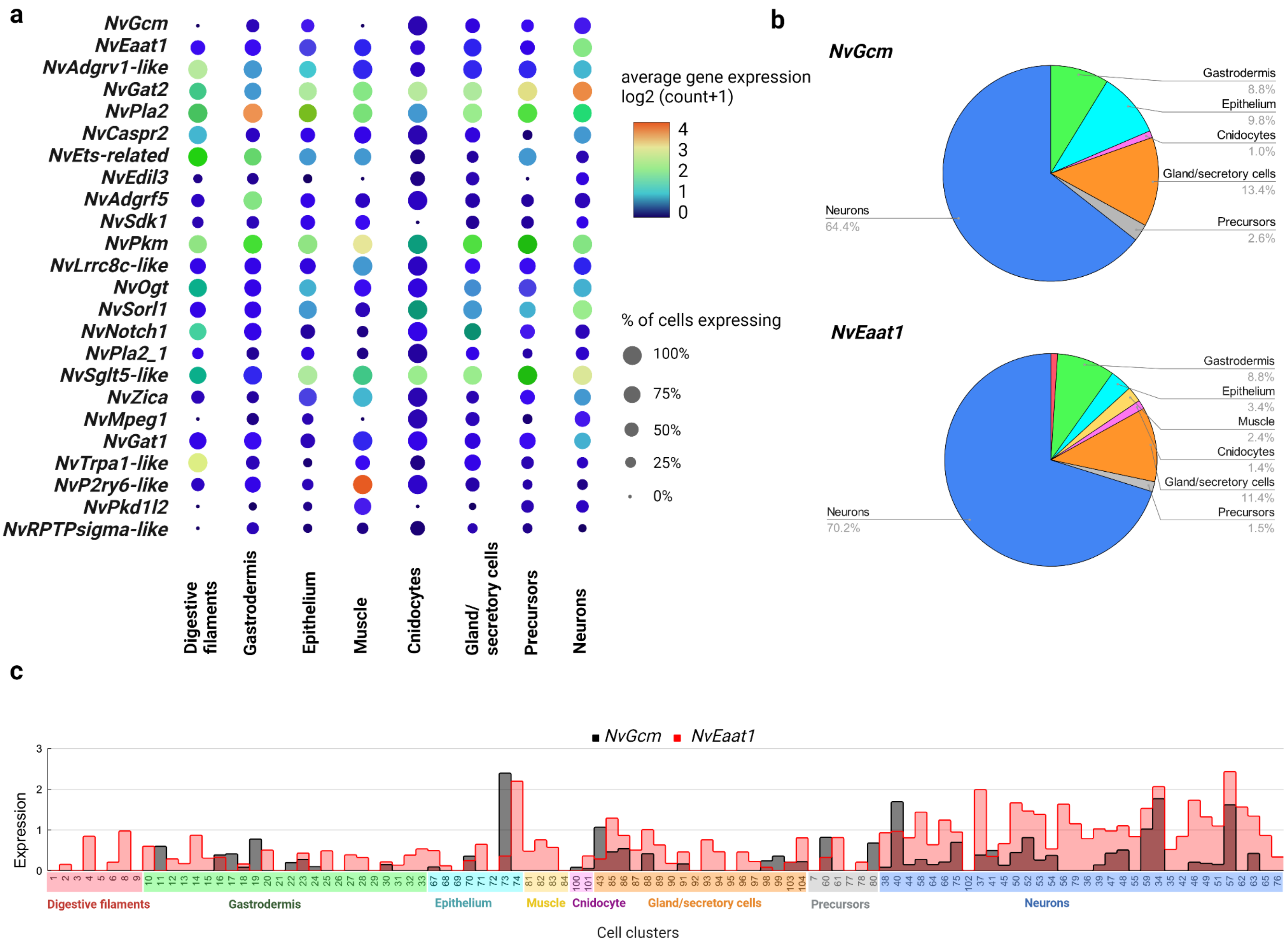
a
NvGcm
NvEaat1
NvAdgrv1-like
NvGat2
NvPla2
NvCaspr2
NvEts-related
NvEdil3
NvAdgrf5
NvSdk1
NvPkm
NvLrrc8c-like
NvOgt
NvSorl1
NvNotch1
NvPla2_1
NvSglt5-like
NvZica
NvMpeg1
NvGat1
NvTrpa1-like
NvP2ry6-like
NvPkd1l2
NvRPTPsigma-like
Digestive filaments
Gastrodermis
Epithelium
Muscle
Cnidocytes
Gland/ secretory cells
Precursors
Neurons
average gene expression log2 (count+1)
4
3
2
1
0
% of cells expressing
100%
75%
50%
25%
0%
b
NvGcm
Gastrodermis 8.8%
Epithelium 9.8%
Cnidocytes 1.0%
Gland/secretory cells 13.4%
Precursors 2.6%
Neurons 64.4%
NvEaat1
Gastrodermis 8.8%
Epithelium 3.4%
Muscle 2.4%
Cnidocytes 1.4%
Gland/secretory cells 11.4%
Precursors 1.5%
Neurons 70.2%
c
NvGcm
NvEaat1
Expression
3
2
1
0
Digestive filaments
Gastrodermis
Epithelium
Muscle
Cnidocyte
Gland/secretory cells
Precursors
Neurons
Cell clusters

Supplementary Figure 3. Expression of *NvGcm* and genes affected by *NvGcm* KD. (a) Average normalized expression of the genes across cell types and the percentage of cell clusters within each cell type expressing the genes in adult animals is shown. For normalization, the raw UMI count of each gene was divided by the total detected RNAs in each cell cluster, multiplied by a scale factor (10,000), a pseudo-count (1) was then added, and the result was log2 transformed. (b) Distribution of *NvGcm* and *NvEaat1* expression across cell types in adult animals: both genes are predominanty expressed in neurons. (c) Normalized expression of *NvGcm* and *NvEaat1* across cell clusters in adult animals are shown.

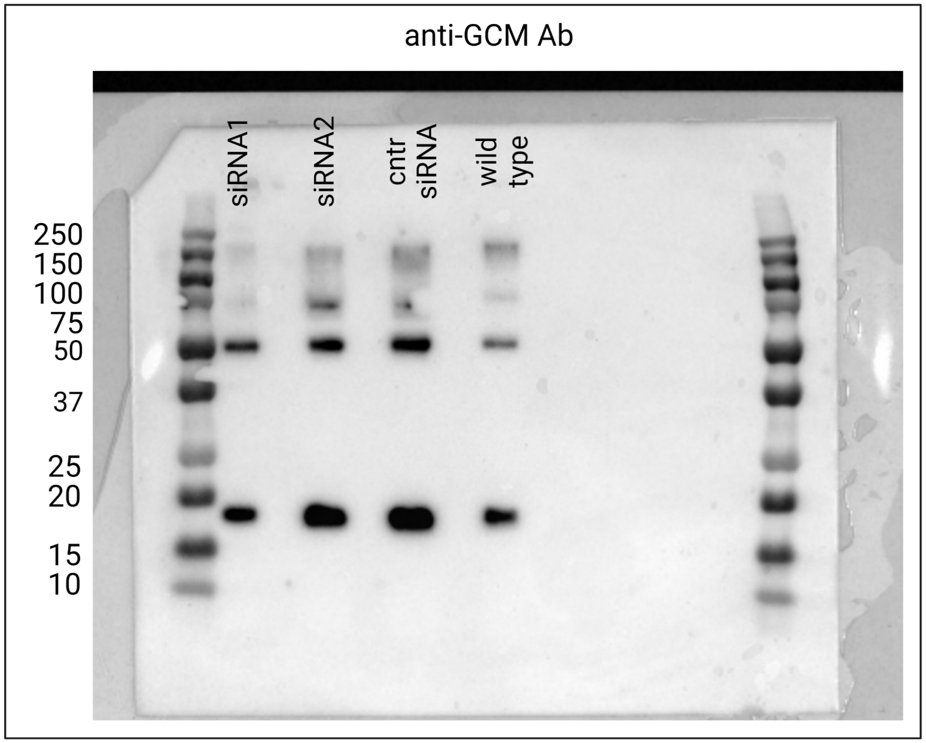

Supplementary Figure 4. An antibody generated specifically for NvGCM lacks specificity producing multiple bands on Western blots. NvGCM-specific siRNA knockdown did not change the band pattern also suggesting the lack of antibody specificity. Western blots were probed with the antibody 1:1000 dilution.

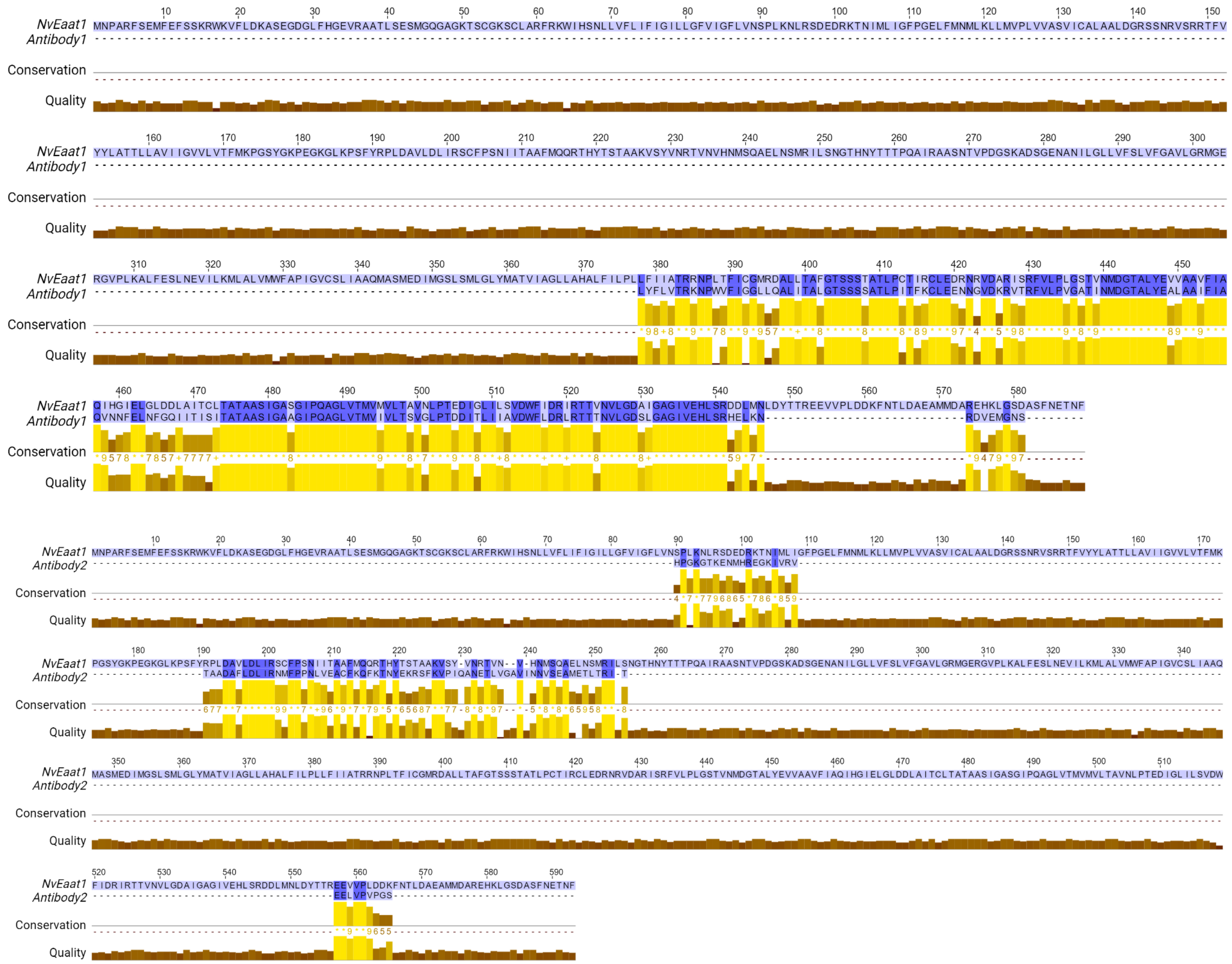

Supplementary Figure 5. Alignment of *Nematostella* EAAT1 and epitope sequences of two antibodies. Antibody 1 is anti-Eaat1 antibody by German Research Products; Antibody 2 is anti-human EAAT1/Glast-1 antibody by Bio-Techne.The sequences were aligned in the T-coffee program with the structural alignment (Espresso) method (Di Tommaso et al., 2011). Image created with JalView Version 2 (Waterhouse et al., 2009).

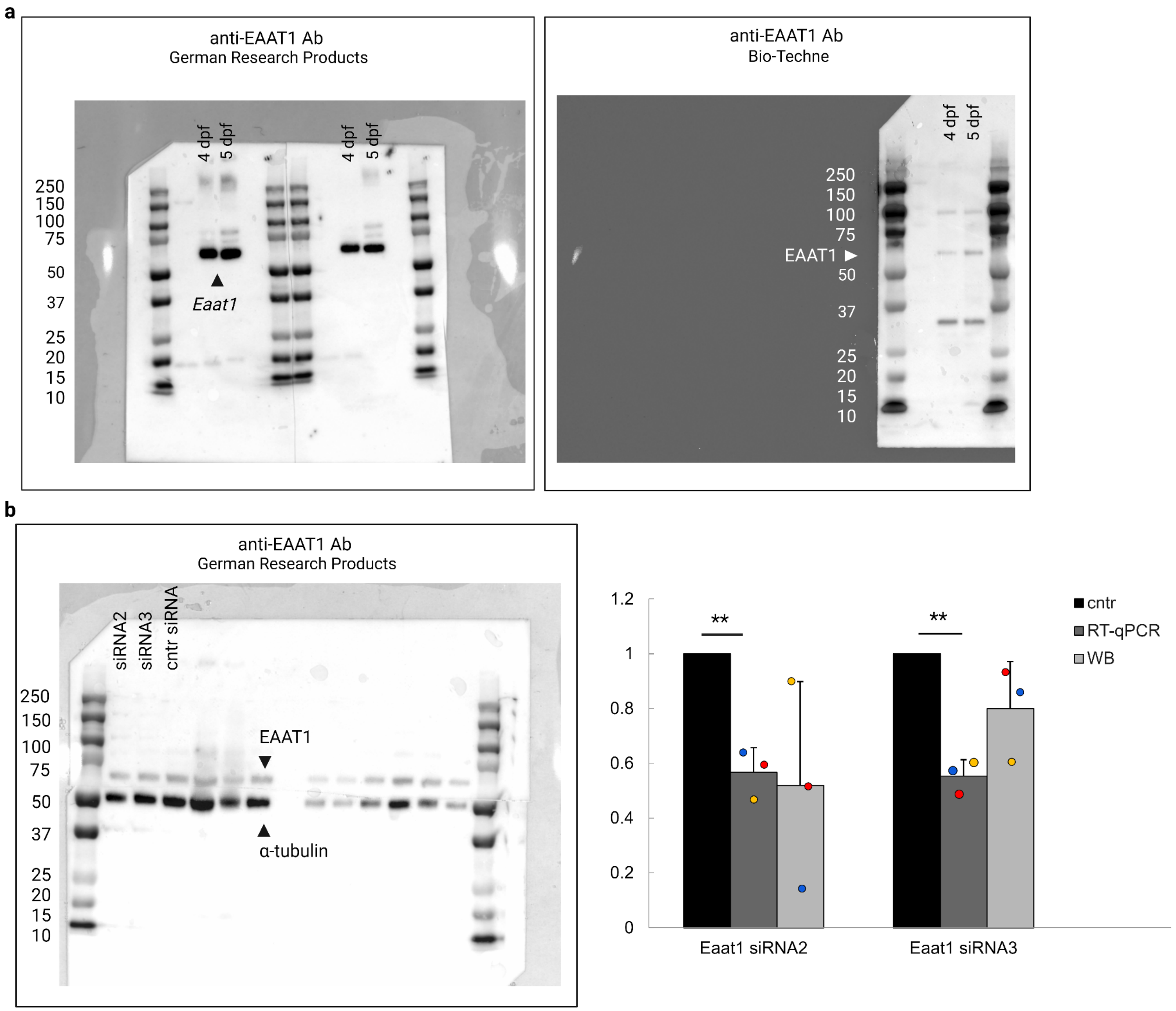

Supplementary Figure 6. Commercially available Anti-EAAT1 antibodies seem to recognize NvEAAT1. (a) Western blots probed with German Research Products anti-EAAT1 antibody (left) and Bio-Techne anti-EAAT1 antibody (right). Specific bands of ~65 KDa are detected with both antibodies as expected based on the *Nematostella* EAAT1 protein sequence. **(b)** Left: Western blot with a loading control (*a-tubulin*) to compare the relative expression of NvEAAT1 in KD vs. control samples probed with German Research Products anti-EAAT1 antibody. Right: expression change (fold change) of *NvEaat1* following KD analyzed by RT-qPCR and WB. Data are given mean ±SD, N=3, **p < 0.01, unpaired t-test.

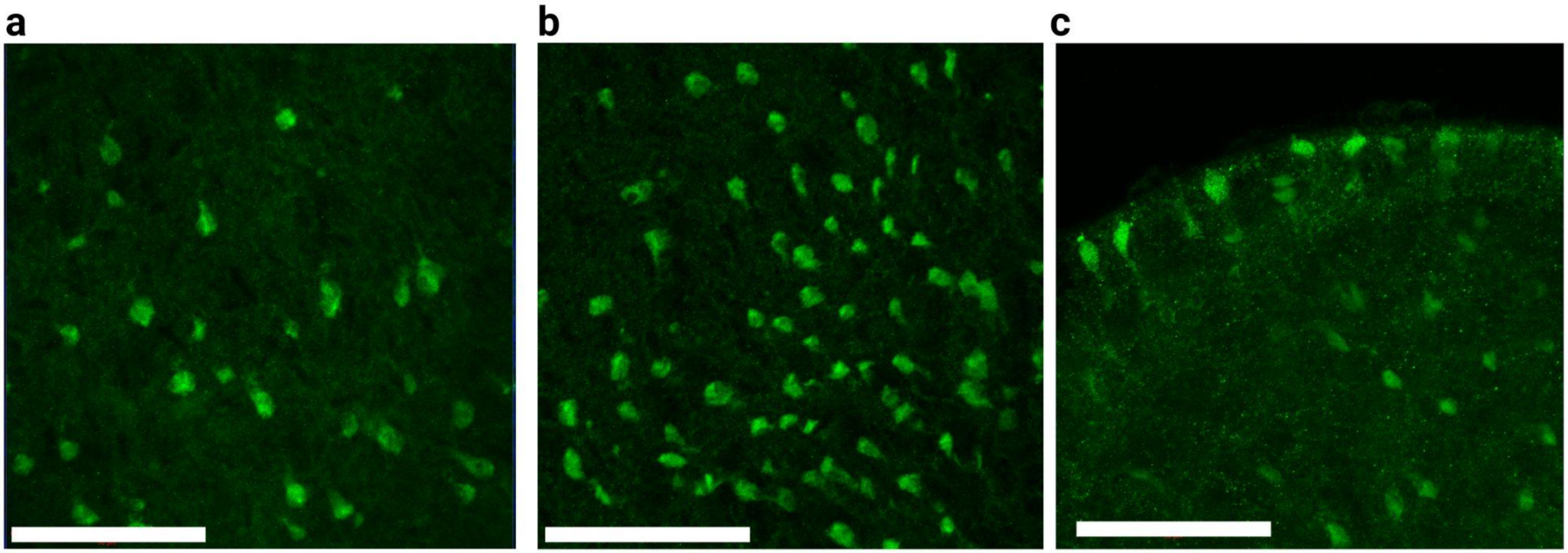

Supplementary Figure 7. Morphology of NvEAAT1-positive cells. EAAT1-expressing cells stained with German Research Products (rabbit) anti-EAAT1 antibody. Planula stage (5dpf) (a,b) Aboral region (c) Apical tissue. Scale bar: 50 µm.

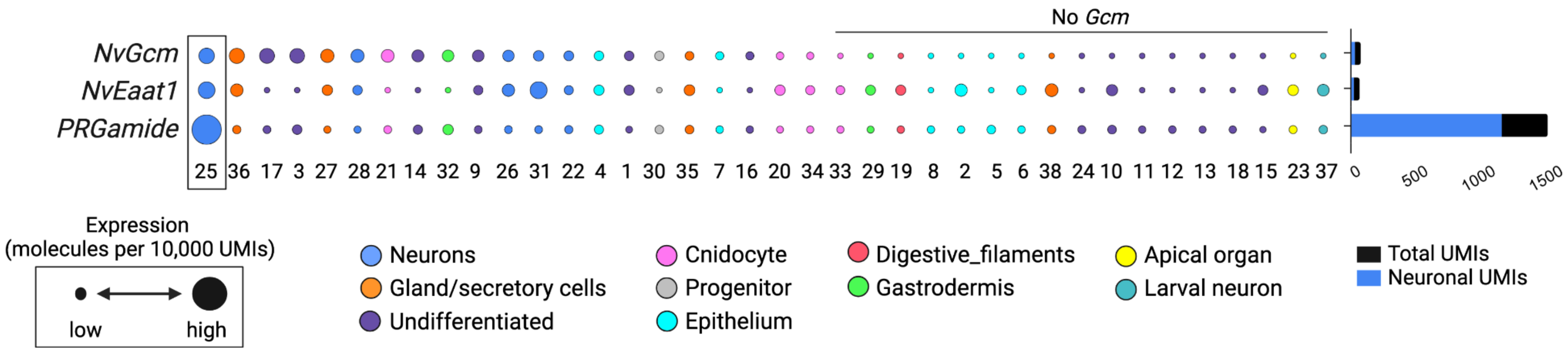

Supplementary Figure 8. Expression of *NvGcm*, *NvEaat1* and PRGamide across larval cell clusters in *N.vectensis*. Expression of the genes is based on scRNA-seq data (Sebé-Pedrós et al. 2018). The dot plot shows the normalized expression values (molecules per 10,000 UMIs) scaled by the gene. Dot size from smallest to largest corresponds to the lowest and highest expression, respectively. Cell clusters are organized left to right in the descending order of *NvGcm* expression.

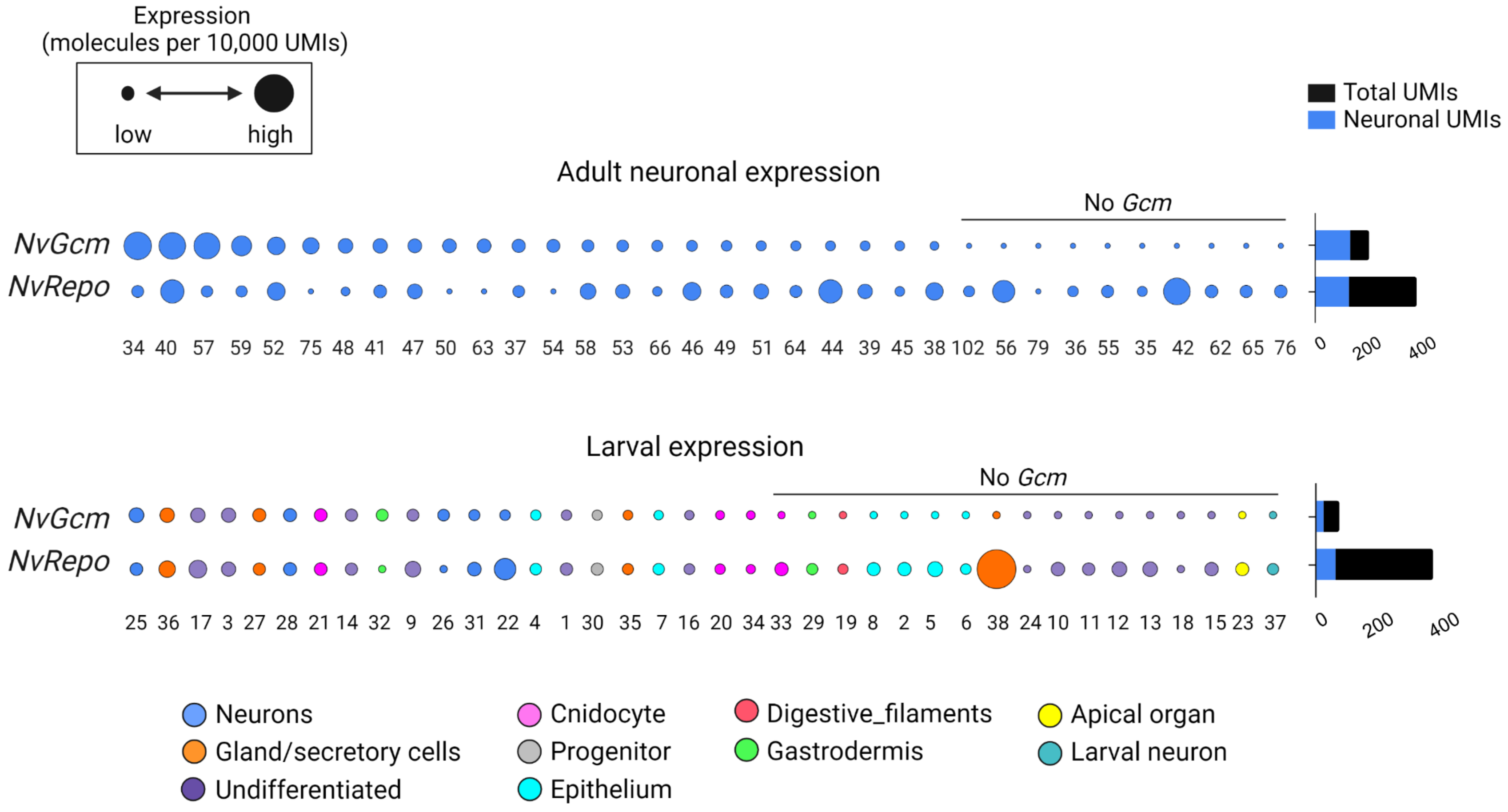

Supplementary Figure 9. Expression of *NvRepo* and *NvGcm* across neuronal cell clusters in adult animals (top) and all the larval cell clusters (bottom). Expression of the genes is based on scRNA-seq data (Sebé-Pedrós et al. 2018). The dot plot shows the normalized expression values (molecules per 10,000 UMIs) scaled by the gene. Dot size from smallest to largest corresponds to lowest and highest expression, respectively. Cell clusters are organized left to right in the descending order of *NvGcm* expression.

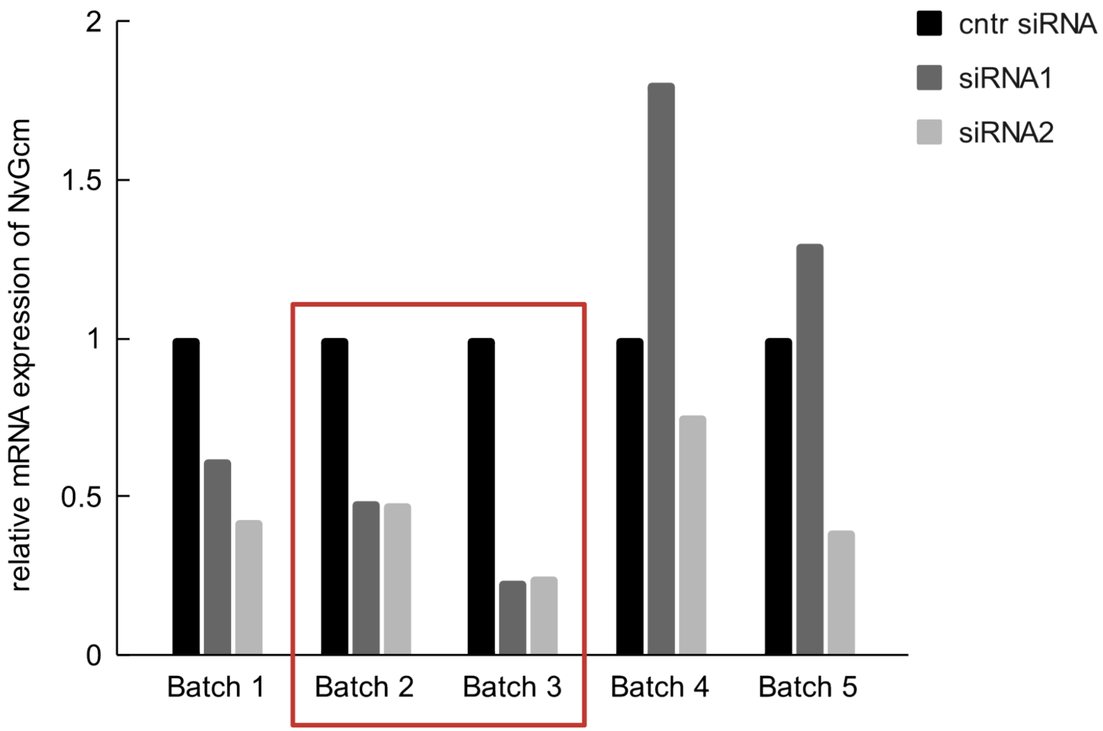

Supplementary Figure 10. KD efficiency of *Gcm*-specific siRNAs. KD efficiency of *Gcm*-specific siRNA1 and siRNA2 in batches used for RNA-seq. Batch 2 and Batch 3 (framed in red) were selected for DE gene analysis.

Supplementary Table 1. Species used for GCM phylogenetic analysis. Sequences were taken from Genbank.

Phylum	Species	Gene name, Accession ID
Chordata	*Homo sapiens*	GCM1, EAX04412.1
		GCM2, NP004743
	Mus musculus	GCM1, EDL26359.1
		GCM2, NP_032130
	Gallus gallus	GCM1, AAR24261
		GCM2, BAD72825
	Danio rerio	GCM2, AAI62304
Hemichordata	*Saccoglossus kowalevskii*	GCM, NP_001161553
Cephalochordata	*Branchiostoma floridae*	GCM, XP_002z591781
Arthropoda	*Drosophila melanogaster*	GCM1, AAC46912
	Drosophila melanogaster	GCM2, NP_609302
	Tribolium castaneum	GCM, EFA04430
Mollusca	*Lottia gigantea*	GCM, XP_009048823
	Biomphalaria glabrata	GCM, XP_013078709
	Octopus bimaculoides	GCM, XP_014777859
	Crassostrea gigas	GCM, EKC24558
Annelida	*Platynereis dumerilii*	GCM, CCK33024
Platyhelminthes	*Echinococcus granulosus*	GCM, CDS20483

Brachiopoda	*Lingula anatina*	GCM, XP_013399310
Echinodermata	*Echinocardium cordatum*	GCM, BAS66823
Tardigrada	*Ramazzottius varieornatus*	GCM, GAU98972
Cnidaria	*Acropora digitifera*	GCM, XP_015774704
	Exaiptasia pallida	GCM, KXJ08668
	Nematostella vectensis	GCM, EDO25565
Fungi	*Coprinopsis cinerea okayama*	Hypothetical protein, EAU87004
	Melampsora larici-populina	Hypothetical protein, EGG12299

Supplementary Table 2. GO terms and function (UniProt) of DE genes grouped by p-adj. Genes used for RT-qPCR verification are highlighted in yellow.

**P-adj< 0.05, downregulated**			
**Nv_vienna_ID**	**NCBI_accession**	**Function /BLAST hit**	**GO/KEGG pathway**
NVE38	XM_001641976	pyruvate kinase PKM. PKM2 inhibition increases substrate flux through the pentose phosphate pathway to generate reducing equivalents (NADPH and GSH) and protect against oxidative stress.	carbohydrate/energy metabolism
NVE22599	XM_032367192	uncharactarized, blastp - no hit	uncharacterized/cnidaria-specific
NVE21209	XM_032376191	uncharactarized, blastp - no hit	uncharacterized/cnidaria-specific
NVE11899	XM_001641371	uncharactarized, blast hit - [Acropora] TNF receptor-associated factor 5-like	uncharacterized/cnidaria-specific
NVE6277	XM_032376361	collagen alpha-4 chain, integrin-like	ECM/cell adhesion
NVE13045	XM_032366926	uncharactarized, blastp - no hit	uncharacterized/cnidaria-specific
NVE14657	XM_032376191	uncharactarized, blastp - no hit	uncharacterized/cnidaria-specific
NVE12839	XM_032366921	uncharactarized, blastp - no hit	uncharacterized/cnidaria-specific
NVE17933	XM_032370086	uncharactarized, blastp - no hit	uncharacterized/cnidaria-specific
NVE7172	XM_032371668	uncharactarized, blastp - no hit	uncharacterized/cnidaria-specific
NVE12840	XM_032366924	uncharactarized, blastp - no hit	uncharacterized/cnidaria-specific
NVE13565	XR_004291976	ncRNA	ncRNA
NVE24747	XR_004296070	misc_RNA	misc_RNA
NVE4321	XM_032377551	supwaprin-a (antimicrobial)	immune response
NVE13564	XM_032387386	sortilin-related receptor, neuronal apolipoprotein E receptor, the gene for which is predominantly expressed in the central nervous system	lipid metabolism
NVE22531	XM_032375707	uncharactarized, blastp - no hit	uncharacterized/cnidaria-specific

NVE25055	XM_032368708	receptor-type tyrosine-protein phosphatase kappa, blast hit - PTPRD (Receptor-type tyrosine-protein phosphatase delta), can bidirectionally induce pre- and post-synaptic differentiation of neurons by mediating interaction with IL1RAP and IL1RAPL1 trans-synaptically. Involved in pre-synaptic differentiation through interaction with SLITRK2.	protein modification
NVE831	XM_032380069	blast hit - mucin-3A-like isoform X2 [Stylophora pistillata], major glycoprotein component of a variety of mucus gels. Thought to provide a protective, lubricating barrier against particles and infectious agents at mucosal surfaces. May be involved in ligand binding and intracellular signaling.	TF/transcription
NVE24010	none	uncharacterized, blastp - no hit	uncharacterized/cnidaria-specific
NVE24376	XM_032381802	UBX protein domain is found in ubiquitin-regulatory proteins, which are members of the ubiquitination pathway, as well as a number of other ubiquitin-like proteins including FAF-1 (FAS-associated factor 1), required for the progression of DNA replication forks by targeting DNA replication licensing factor CDT1 for degradation	protein modification
NVE16319	XM_032366645	E3 ubiquitin-protein ligase TM129, transmembrane protein, involved in ER-associated protein degradation	protein degradation
NVE3633	XM_032366195	uncharactarized, blast hit - Intraflagellar transport protein 172 (coral)	uncharacterized/cnidaria-specific
NVE22208	XM_032369057	uncharactarized, blast - no hit	uncharacterized/cnidaria-specific
NVE3634	none	uncharactarized, blast hits - hypothetical proteins in corals	uncharacterized/cnidaria-specific
NVE3381	XR_004294124	ncRNA	ncRNA
NVE14629	XM_032371431	uncharactarized, blast - hypothetical proteins in corals	uncharacterized/cnidaria-specific
NVE5822	XM_032367471	uncharactarized, blast hit - E3 ubiquitin-protein ligase DZIP3 [Exaiptasia pallida], mediates ubiquitination and subsequent proteasomal degradation of target proteins	protein degradation
NVE2774	XM_032375707	uncharactarized, blast - no hit	uncharacterized/cnidaria-specific
NVE830	XM_032380063	contactin-associated protein-like 2 (CNTNAP2)	ECM/cell adhesion

NVE18730	XM_032384910	blast hit - Polycystic kidney disease protein 1-like 3 [Exaiptasia pallida], Component of a calcium channel. May act as a sour taste receptor by forming a calcium channel with PKD1L3 in gustatory cells	Membrane transport
NVE15740	XM_032372064	uncharactarized, blast hits - hypothetical proteins from corals and molluscs	uncharacterized/cnidaria-specific
NVE2552	XM_032368045	Integrase zinc core domain, blast hit - TNF receptor-associated factor 3 [Stylophora pistillata] an essential constituent of several E3 ubiquitin-protein ligase complexes. May have E3 ubiquitin-protein ligase activity and promote 'Lys-63'-linked ubiquitination of target proteins.	Protein synthesis/processing/ degradation
NVE8261	XM_032366377	Neurogenic locus notch homolog protein 1 and Notch 2	Notch signaling
NVE10736	XM_032372378	blast hit - Transposon Ty3-G Gag-Pol polyprotein [Exaiptasia pallida]	uncharacterized/cnidaria-specific
NVE141	XM_032367644	ADGRL4, adhesion G protein-coupled receptor L4, Endothelial orphan receptor that acts as a key regulator of angiogenesis	ECM/Cell adhesion
NVE13473	XM_032367885	Replication factor C subunit 1. The elongation of primed DNA templates by DNA polymerase delta and epsilon requires the action of the accessory proteins PCNA and activator 1. This subunit binds to the primer-template junction. Binds the PO-B transcription element as well as other GA rich DNA sequences. Could play a role in DNA transcription regulation as well as DNA replication and/or repair.	DNA replication/repair
NVE8653	AB231867	TF, zinc finger protein ZIC4 (Zinc finger protein of the cerebellum 4) is involved in neurogenesis	TFs/transcription
NVE5670	XM_032376697	sushi, von Willebrand factor type A, EGF and pentraxin domain-containing protein 1 (SVEP1), May play a role in the cell attachment process, multi-domain cell adhesion protein, homologous to the mouse polydom protein, which has been shown to mediate cell-cell adhesion in an integrin dependent-manner in osteogenic cells	ECM/Cell adhesion
NVE16480	XM_032382916	blast hit - E3 ubiquitin-protein ligase DZIP3 [Exaiptasia pallida]	protein degradation
NVE26003	XM_032380640	uncharacterized	uncharacterized/cnidaria-specific
NVE12641	XM_032372363	vicilin-like seed storage protein At2g18540, Seed storage protein.	TFs/transcription
NVE13485	XM_032372265	uncharacterized, blast hit - ATP-binding cassette sub-family D member 3 [Labeo rohita]	uncharacterized/cnidaria-specific

NVE18507	XM_032366926	uncharactarized, blastp hit - none	uncharacterized/cnidaria-specific
NVE13487	XM_032375749	uncharacterized, blast hit - hypothetical protein [Nostoc sp. T09]	uncharacterized/cnidaria-specific
NVE7065	XM_001627778	uncharacterized, TSP1 superfamily domain, blast hit - cell surface glycoprotein 1 [Exaiptasia pallida]	ECM/cell adhesion
NVE7280	XM_032375568	arginine and glutamate-rich protein 1-A, is required for the oestrogen-dependent expression of ESR1 target genes. It functions in cooperation with MED1	TFs/transcription
NVE18121	XR_004295795	ncRNA	ncRNA
NVE9285	none	uncharacterized, blast - no hit	uncharacterized/cnidaria-specific
NVE20550	XM_032369412	EGF-like repeat and discoidin I-like domain-containing protein 3. EDIL3 is a glycoprotein that is secreted by endothelial cells and can associate with the endothelial cell surface and the extracellular matrix.	ECM/cell adhesion
NVE10760	XM_032373473	cell wall protein DAN4-like, DAN4 in yeast is a component of the cell wall	membrane transport
NVE13391	XM_032372047	uncharacterized, Corresponds to Merops family A17. These proteins are homologous to aspartic proteinases encoded by retroposons and retroviruses.	RT/retrotransposons
NVE8561	XM_032374855	organic cation transporter protein (OCTN1). An organic cation transport protein mediates the transport of organic cations across the cell membrane. These proteins are members of the solute carrier family, subfamily 22.	membrane transport
NVE18120	XR_004296913, XM_032385195	ncRNA	ncRNA
NVE444	XM_001631508	YTH domain-containing protein 1, Regulator of alternative splicing that specifically recognizes and binds N6-methyladenosine (m6A)-containing RNAs, directs different complexes to regulate RNA signaling pathways, such as RNA metabolism, RNA splicing, RNA folding, and protein translation.	RNA splicing
NVE9694	XM_032374129	peroxisomal N(1)-acetyl-spermine/spermidine oxidase, Flavoenzyme which catalyzes the oxidation of N1-acetylspermine to spermidine and is thus involved in the polyamine back-conversion	lipid metabolism
NVE18446	XM_032375707	uncharactarized, blast - no hit	uncharacterized/cnidaria-specific
NVE3596	XM_032377969	Sorting nexin-13, RGS-PX1 is a member of both the regulator of G protein signaling (RGS) and sorting nexin (SNX) protein families.	Protein synthesis/processing/ degradation

NVE22605	XM_032376127	blast hit - extensin-like mRNA, extensins are a family of flexuous, rodlike, hydroxyproline-rich glycoproteins (HRGPs) of the plant cell wall or zonadhesin (salmon match). This gene encodes a protein that functions in the species specificity of sperm adhesion to the egg zona pellucida. The encoded protein is located in the acrosome and may be involved in signaling or gamete recognition.	TFs/transcription
NVE10366	XM_032362710	uncharacterized, blast hits - uncharacterized proteins in cnidaria and molluscs	uncharacterized/cnidaria-specific
NVE12411	XM_032372478	bifunctional polynucleotide phosphatase/kinase, Plays a key role in the repair of DNA damage, functioning as part of both the non-homologous end-joining (NHEJ) and base excision repair (BER) pathways.	DNA replication/repair
NVE24235	XM_001636600	DNA replication ATP-dependent helicase/nuclease DNA2, Key enzyme involved in DNA replication and DNA repair in nucleus and mitochondria.	DNA replication/repair
NVE1600	XR_004293153	ncRNA	ncRNA
NVE7392	XM_001620713	cilia- and flagella-associated protein 74, as part of the central apparatus of the cilium axoneme may play a role in cilium movement.	cell motility
NVE18180	XM_032371133	adhesin BpaC-like, cell adhesion protein identified in bacteria	ECM/cell adhesion
NVE12293	XM_032372590	blast hit - cilia- and flagella-associated protein 99 [Delphinapterus leucas]	cell motility
NVE9769	XM_032367534	uncharacterized, blast hit - Alpha-1,6-mannosylglycoprotein 6-beta-N-acetylglucosaminyltransferase A [Exaiptasia pallida], Catalyzes the addition of N-acetylglucosamine (GlcNAc) in beta 1-6 linkage to the alpha-linked mannose of biantennary N-linked oligosaccharides, Via its role in the biosynthesis of complex N-glycans, plays an important role in the activation of cellular signaling pathways, reorganization of the actin cytoskeleton, cell-cell adhesion and cell migration.	ECM/cell adhesion
NVE22455	XM_032365909	polycystic kidney disease protein 1-like 2, may function as an ion-channel regulator, involved in cell-cell/matrix interactions. May function as a G-protein-coupled receptor.	membrane transport
NVE24871	XM_032381419	Cartilage matrix protein-like, is a major component of the extracellular matrix of non-articular cartilage. It binds to collagen.	ECM/cell adhesion
NVE12227	XM_032386874	uncharacterized	uncharacterized/cnidaria-specific
NVE7553	XM_032387267	reverse transcriptase	RT/retrotransposons
NVE7577	XM_032375405	heterogeneous nuclear ribonucleoprotein L-like (HNRNPLL), RNA-binding protein that functions as regulator of alternative splicing for multiple target mRNAs	RNA splicing

NVE21596	XM_032383320	probable ATP-dependent RNA helicase DDX10 (RNA helicase).	RNA splicing
NVE19901	XR_004292618	misc_RNA	misc_RNA
NVE13277	XM_032372150	potassium channel subfamily T member 1 (voltage-gated ion channels), outwardly rectifying potassium channel subunit that may coassemble with other Slo-type channel subunits. Activated by high intracellular sodium or chloride levels. Activated upon stimulation of G-protein coupled receptors, such as CHRM1 and GRIA1.	membrane transport
NVE24988	XR_004295998	misc_RNA	misc_RNA
NVE17173	XM_032369781	von Willebrand factor D and EGF domain-containing protein (calcium ion binding)	ECM/cell adhesion
NVE16596	XR_004297107	ncRNA, blast hit - hypothetical proteins (Cnidaria)	ncRNA
NVE7066	XM_001627778	same as NVE7065	ECM/cell adhesion
NVE4276	XM_032362635	arginine and glutamate-rich protein 1-A (ARGLU1)	TFs/transcription
NVE20392	XM_032369454	zinc finger C3H1 domain-containing protein	TFs/transcription
NVE14680	XR_004295795	ncRNA, miscRNA	ncRNA
NVE16225	XM_001623467	bZIP transcription factor-like	TFs/transcription
NVE8051	XM_032367102	sushi, von Willebrand factor type A, EGF and pentraxin domain-containing protein 1 (SVEP1), May play a role in the cell attachment process.	ECM/cell adhesion
NVE7655	XM_032375303	predicted, blast hits - uncharacterized proteins in Chidaria, REJ domain - domain is found in PKD1 and the sperm receptor for egg jelly. The function of this domain is unknown. IPT/TIG domain. This family consists of a domain that has an immunoglobulin-like fold. These domains are found in cell surface receptors such as Met and Ron as well as in intracellular transcription factors where it is involved in DNA binding.	membrane transport
**p-adj < 0.05, upregulated**			
NVE17843	XR_004296956	miscRNA	miscRNA
NVE14448	XM_032371523	uncharacterized	uncharacterized/cnidaria-specific
NVE5252	XM_032368149	uncharacterized, blast hit- galactose-specific lectin [Anthopleura japonica]	uncharacterized/cnidaria-specific
NVE14983	XM_032371282	60S ribosomal protein L15	ribosomal

NVE4717	XM_032364190	uncharacterized, blast hit - Pancreatic secretory granule membrane major glycoprotein GP2 [homo sapiens], LZP variant II [Mus musculus], Zona pellucida (ZP) domain has been recognized in a number of receptor-like eukaryotic glycoproteins, which is involved in many important biological processes, such as signal transduction, development, differentiation.	ECM/cell adhesion
NVE10004	XM_001626357	adhesion G protein-coupled receptor B1 (ADGRB1 or BAI1), Phosphatidylserine receptor which enhances the engulfment of apoptotic cells.	ECM/cell adhesion
NVE3218	XM_032378343	macrophage-expressed gene 1 protein, Macrophage Expressed Gene-1 (MPEG-1; also termed Perforin-2) is an endosomal / phagolysosomal perforin-like protein that is conserved across the metazoan kingdom and that functions within the phagolysosome to damage engulfed microbes. Plays a key role in the innate immune response following bacterial infection by inserting into the bacterial surface to form pores	immune response
NVE23561	XM_032382138	PLA2_1 phospholipase A2 A2-actitoxin-Cgg2a, involved in Lipid metabolism (cleaves fatty acids), phospholipase A2 (PLA2), membrane associated precursor [Homo sapiens], Ca2+-requiring, secretory enzymes that have been implicated in a number of biological processes, such as modification of eicosanoid generation, inflammation, host defense, and atherosclerosis.	lipid metabolism
NVE1644	XM_032379490	uncharacterized	uncharacterized/cnidaria-specific
NVE12742	XM_032385880	uncharacterized	uncharacterized/cnidaria-specific
NVE2195	XM_032364690	blast - galaxin-like, Galaxin, which encodes for a matrix protein suspected to be involved in calcification, is originally identified from the coral. It is hypothesized that the coral galaxin homologs are recruited as biomineralization proteins when Scleractinia diverged from non-biomineralizing taxa during the Triassic56 because galaxin-like proteins are also found in non-calcifying taxa outside Cnidaria	uncharacterized/cnidaria-specific
NVE9883	XM_032362934.1	uncharactarized, blast hit - ETS-related transcription factor Elf-1	TFs/transcription
NVE23912	XM_032365681	uncharactarized	uncharacterized/cnidaria-specific
**p-adj < 0.1, downregulated**			
NVE2938	XM_001639423	calcineurin-binding protein cabin-1. May be required for replication-independent chromatin assembly. May serve as a negative regulator of T-cell receptor (TCR) signaling via inhibition of calcineurin. Inhibition of activated calcineurin is dependent on both PKC and calcium signals. Acts as a negative regulator of p53/TP53 by keeping p53 in an inactive state on chromatin at promoters of a subset of it's target genes.	TFs/transcription
NVE18895	XM_032366377	neurogenic locus notch homolog protein 1	Notch signaling

NVE23097	XR_004295937	misc_RNA	misc_RNA
NVE21821	XM_032362584	bromodomain-containing protein 7 - is a transcription regulator that is normally highly expressed in the testis, Acts both as coactivator and as corepressor. May play a role in chromatin remodeling. Activator of the Wnt signaling pathway in a DVL1-dependent manner by negatively regulating the GSK3B phosphotransferase activity.	TFs/transcription
NVE2281	XM_032379039	uncharacterized, blast hit - transient receptor potential protein 5 [Mus musculus, homo sapiens], receptor-activated non-selective calcium permeant cation channel, transient receptor potential 5 TRPC5 (also TrpC5, trp-5 or trp5) is one of the seven mammalian TRPC proteins. Its known functional property is that of a mixed cationic plasma membrane channel with calcium permeability.	membrane transport
NVE5335	DQ518750, BR000683 , XM_032362962	Mox gene cluster, gene for opsin (match - melanopsin [mus musculus]), Photoreceptor required for regulation of circadian rhythm. Contributes to pupillar reflex and other non-image forming responses to light. May be able to isomerize covalently bound all-trans retinal back to 11-cis retinal.	TFs/transcription
NVE25263	XM_001640126	proton channel OtopLc. Proton-selective channel that specifically transports protons into cells. Proton-selective channel activity is probably required in cell types that use changes in intracellular pH for cell signaling or to regulate biochemical or developmental processes.	membrane transport
NVE1719	XM_032379414	piwi-like protein 1, Endoribonuclease that plays a central role in postnatal germ cells by repressing transposable elements and preventing their mobilization, which is essential for the germline integrity.	RT/retrotransposons
NVE3923	XR_004293389	ncRNA	ncRNA
NVE13370	XM_032372074	RT-like family, DUF5641 (protein domain family of unknown function).	RT/retrotransposons
NVE13426	XM_001624701	formin-binding protein 4, protein containing two tryptophan-rich WW domains that binds the proline-rich formin homology 1 domains of formin family proteins, suggesting a role in the regulation of cytoskeletal dynamics during cell division and migration.	cell motility
NVE1653	XM_032379482	transient receptor potential cation channel subfamily A member 1, Receptor-activated non-selective cation channel involved in pain detection and possibly also in cold perception, oxygen concentration perception, cough, itch, and inner ear function.	membrane transport
NVE24012	XM_032365673	leukocyte receptor cluster member 8 (LENG8), signal transduction, genes involved in blood-brain barrier (BBB) formation and integrity (Schiavone et al, 2017), ubiquitous expression	membrane transport

NVE25595	XM_032380977	tetratricopeptide repeat protein 12, The TTC12 gene encodes a protein containing several domains known to mediate protein-protein interactions and the assembly of multiprotein complexes. TTC12 is believed to play a role in the assembly of dynein arms in motile cilia in both human respiratory cells and sperm flagella	Protein synthesis/processing/degradation
NVE5337	XM_001628627	uncharacterized, blast hits - hypothetical proteins	uncharacterized/cnidaria-specific
NVE14627	XM_032371433	uncharacterized, blast hits - hypothetical proteins	uncharacterized/cnidaria-specific
NVE4725	XM_032364135	uncharacterized, RT, retrotransposon peptidase, zinc binding domain, retrotransposon	RT/retrotransposons
NVE24441	XM_032385326	uncharacterized, blast hits - hypothetical proteins	uncharacterized/cnidaria-specific
NVE9889	XM_032362858	DNA excision repair protein ERCC-6-like 2. This gene encodes a member of the Snf2 family of helicase-like proteins. The encoded protein may play a role in DNA repair and mitochondrial function.	DNA replication/repair
NVE1830	XM_032366645	E3 ubiquitin-protein ligase TM129, same as NVE16319	Protein synthesis/processing/degradation
NVE21065	XM_032366926	uncharacterized	uncharacterized/cnidaria-specific
NVE25845	XM_032375707	uncharacterized, same as NVE22531	uncharacterized/cnidaria-specific
NVE25715	XM_032366922	uncharacterized, blast hit - LysM peptidoglycan-binding domain-containing protein [Moraxella sp.]	uncharacterized/cnidaria-specific
NVE8970	XM_032386874	uncharacterized, blast hit - zinc-finger DNA-binding protein [Operophtera brumata]	TFs/transcription
NVE10940	XM_032373417	adhesion G-protein coupled receptor D1-like (ADGRD1). Orphan receptor. Signals via G(s)-alpha family of G-proteins (PubMed:22025619, PubMed:22575658). Has protumorigenic function especially in glioblastoma (PubMed:27775701). Plays a role in cell-cell adhesion and neuron guidance via its interactions with FLRT2 and FLRT3 that are expressed at the surface of adjacent cells (PubMed:26235030).	ECM/cell adhesion
NVE20529	XM_032373468	uncharacterized, protein unc-13 homolog C-like [Pocillopora damicornis]. May play a role in vesicle maturation during exocytosis as a target of the diacylglycerol second messenger pathway.	endocytosis/exocytosis
NVE7559	XM_032370283	uncharacterized, blast - no hits	uncharacterized/cnidaria-specific
NVE23107	XM_032381141	uncharacterized, blast - no hits	uncharacterized/cnidaria-specific
**p-adj < 0.1, upregulated**			

NVE8459	XM_032374949	glycine-rich RNA-binding protein 7, Plays a role in RNA transcription or processing during stress. Binds RNAs and DNAs sequence with a preference to single-stranded nucleic acids. Displays strong affinity to poly(U) and poly(G) sequence. Involved in mRNA alternative splicing of numerous targets by modulating splice site selection.	RNA splicing
NVE13796	XM_032371808	polycystic kidney disease protein 1-like 2, May function as an ion-channel regulator. May function as a G-protein-coupled receptor.	membrane transport
NVE3046	XM_001639565	glutamic acid-rich protein	TFs/transcription
**p-adj < 0.2, upregulated**			
NVE8526	XM_001627177	uncharacterized, blast - no hits	uncharacterized/cnidaria-specific
NVE17842	XM_032385402	uncharacterized, blast - no hits	uncharacterized/cnidaria-specific
NVE14450	XM_032371522	uncharacterized, galactose-specific lectin [Anthopleura japonica]	ECM/cell adhesion
NVE8043	XM_001641744	40S ribosomal protein S23	ribosome
NVE11578	XM_001620758	40S ribosomal protein S9	ribosome
NVE9450	XM_001638285	60S ribosomal protein L30	ribosome
NVE12548	XM_032361981	tetratricopeptide repeat protein 28-like, TTC28, During mitosis, may be involved in the condensation of spindle midzone microtubules, leading to the formation of midbody	Protein synthesis/processing/degradation
NVE2256	XM_001630433	60S ribosomal protein L34	ribosome
NVE2295	XM_001630427	60S ribosomal protein L42-A	ribosome
NVE7522	XM_032363570	calponin-1, Calponin 1 is a basic smooth muscle protein, The expression of CNN1 is specific to differentiated mature smooth muscle cells, suggesting a role in contractile functions.	cell motility
NVE8334	XM_032363335	neurogenic locus notch homolog protein 1-like	Notch signaling
NVE21167	XM_001634346	LWamide neuropeptides	Neuroactive ligand-receptor
NVE6169	XM_032363930	uncharacterized, blast - Fert2 protein, partial [Mus musculus], Tyrosine-protein kinase that acts downstream of cell surface receptors for growth factors and plays a role in the regulation of the actin cytoskeleton, microtubule assembly, lamellipodia formation, cell adhesion, cell migration and chemotaxis. Plays a role in synapse organization, trafficking of synaptic vesicles, the generation of excitatory postsynaptic currents and neuron-neuron synaptic transmission.	cell motility
NVE9019	XM_032374594	B-cell lymphoma 3 protein (BCL3) Contributes to the regulation of transcriptional activation of NF-kappa-B target genes. In the cytoplasm,	NF-kappa B

		inhibits the nuclear translocation of the NF-kappa-B p50 subunit. In the nucleus, acts as a transcriptional activator that promotes transcription of NF-kappa-B target genes. Contributes to the regulation of cell proliferation	
NVE2142	XM_032364650	beta-ketoacyl-ACP synthase 3 [Nocardioides sp. SLBN-35], Beta-ketoacyl-ACP synthase is found as a component of a number of enzymatic systems, including fatty acid synthetase (FAS)	lipid metabolism
NVE7499	XM_032363508	TNFAIP3-interacting protein 3-like, Binds to zinc finger protein TNFAIP3 and inhibits NF-kappa-B activation induced by tumor necrosis factor, Toll-like receptor 4 (TLR4), interleukin-1 and 12-O-tetradecanoylphorbol-13-acetate. Overexpression inhibits NF-kappa-B-dependent gene expression in response to lipopolysaccharide at a level downstream of TRAF6 and upstream of IKBKB. NF-kappa-B inhibition is independent of TNFAIP3 binding.	NF-kappa B
**p-adj < 0.2, downregulated**			
NVE18073	XM_032385202	sodium/glucose cotransporter 5, High capacity transporter for mannose and fructose and, to a lesser extent, glucose, AMG, and galactose. found in the intestinal mucosa (enterocytes) of the small intestine (SGLT1)	carbohydrate/energy metabolism
NVE10425	XM_032362651	KH homology domain-containing protein 4 - RNA-binding protein involved in pre-mRNA splicing.	RNA splicing
NVE10681	XM_032370200	uncharacterized, blast hits - hypothetical proteins (cnidaria)	uncharacterized/cnidaria-specific
NVE16938	XM_032362591	uncharacterized	uncharacterized/cnidaria-specific
NVE5342	XM_032376858	uncharacterized, RT-like superfamily, peptidase superfamily, DUF1759 unknown function	RT/retrotransposons
NVE17112	XR_004297054	ncRNA	ncRNA
NVE13369	XR_004293601	miscRNA	miscRNA
NVE6128	XM_032363949	uncharacterized, blast hits - other uncharacterized proteins (incl zebrafish)	uncharacterized/cnidaria-specific
NVE5726	XM_032376662	uncharacterized, blast hits - other uncharacterized proteins (cnidaria)	uncharacterized/cnidaria-specific
NVE5672	XM_032376697	sushi, von Willebrand factor type A, EGF and pentraxin domain-containing protein 1 (SVEP1) calcium ion binding and chromatin binding.	ECM/cell adhesion
NVE24253	XM_032369598	uncharacterized, blast hits - other uncharacterized proteins	uncharacterized/cnidaria-specific
NVE18659	XM_032369784	uncharacterized, blast hits - other uncharacterized proteins	uncharacterized/cnidaria-specific
NVE23504	XR_004292166	miscRNA	miscRNA
NVE2560	XR_004293834	ncRNA	ncRNA

NVE10977	XM_032373395	uncharacterized, Reverse transcriptase (RTs) in retrotransposons. This subfamily represents the RT domain of a multifunctional enzyme.	RT/retrotransposons
NVE19504	XM_032369412	EGF-like repeat and discoidin I-like domain-containing protein 3 (same as NVE20550)	ECM/cell adhesion
NVE14525	XM_032386819	flocculation protein FLO11-like, Cell wall protein that participates in adhesive cell-cell interactions during yeast flocculation, a reversible, asexual and Ca2+-dependent process in which cells adhere to form aggregates (flocs) consisting of thousands of cells	ECM/cell adhesion
NVE10424	XM_032362651	KH homology domain-containing protein 4	RNA splicing
NVE9099	XM_032374520	multidrug resistance-associated protein 4, belongs to the ATP-binding cassette (ABC) superfamily of transporters, is localized to the basolateral membrane of hepatocytes and the apical membrane of renal proximal tubule cells.	Membrane transport
NVE22907	XM_032371133	autotransporter adhesin BpaC-like, Autotransporters form a large family of outer membrane proteins specifying diverse biological traits of Gram-negative bacteria	ECM/cell adhesion
NVE20069	XM_032369520	uncharacterized, SDK1, protein sidekick-1-like [Pocillopora damicornis]. Adhesion molecule that promotes lamina-specific synaptic connections in the retina.	ECM/cell adhesion
NVE1287	XM_032365086	thyroglobulin-like, Thyroglobulin (Tg) is a vertebrate secretory protein synthesized in the thyrocyte endoplasmic reticulum (ER)	ECM/cell adhesion
NVE9264	XM_032374374	myosin-11-like - Muscle contraction - It is a major contractile protein, converting chemical energy into mechanical energy through the hydrolysis of ATP.	cell motility
NVE15277	XR_004293291	ncRNA	ncRNA
NVE11894	XR_004295885	ncRNA	ncRNA
NVE6633	XM_032376014	uncharacterized, blast hits - other uncharacterized proteins, incl zn finger protein	uncharacterized/cnidaria-specific
NVE17538	XM_032369784	uncharacterized, blast hits - other uncharacterized proteins, same as NVE18659	uncharacterized/cnidaria-specific
NVE4885	XR_004292165.1	miscRNA	miscRNA
NVE22620	XM_032382832	tetratricopeptide repeat protein 14-like, TTC14, nucleic acid binding, function unknown	uncharacterized/cnidaria-specific
NVE9699	XM_032374134	uncharacterized, blast hits - other hypothetical proteins	uncharacterized/cnidaria-specific
NVE16190	XR_004293155	ncRNA	ncRNA

NVE11097	XM_032369784	same as NVE18659, uncharacterized	uncharacterized/cnidaria-specific
NVE15479	XM_032386450	multidrug resistance-associated protein 4, cystic fibrosis transmembrane conductance regulator [Mus musculus] - The CFTR gene codes for an ABC transporter-class ion channel protein that conducts chloride ions across epithelial cell membranes.	Membrane transport
NVE2545	XR_004295885	ncRNA	ncRNA
NVE20278	XM_032386874	uncharacterized, blast hits - hypothetical proteins	uncharacterized/cnidaria-specific
NVE18894	XM_032366394	neurogenic locus notch homolog protein 1	Notch signaling
NVE13408	XM_032369784	uncharacterized, blast hits -other uncharacterized proteins, same as NVE18659	uncharacterized/cnidaria-specific
NVE17860	XM_032385326	uncharacterized, blast hits - hypothetical proteins of cnidaria and mollusca, same as NVE24441	uncharacterized/cnidaria-specific
NVE253	XM_032367740	uncharacterized, Collagen alpha-1(VII) chain [Exaiptasia pallida], collagen alpha-4(VI) chain isoform X1 [Mus musculus], integrin-like	ECM/cell adhesion
NVE443	XM_032380333	EAAT1 (excitatory amino acid transporter 1)	Membrane transport, neurotransmission
NVE2551	XR_004295429	ncRNA	ncRNA
NVE12193	XM_032362078	adhesion G-protein coupled receptor V1-like, G-protein coupled receptor which has an essential role in the development of hearing and vision. In response to extracellular calcium, activates kinases PKA and PKC to regulate myelination by inhibiting the ubiquitination of MAG, thus enhancing the stability of this protein in myelin-forming cells of the auditory pathway. In retina photoreceptors, the USH2 complex is required for the maintenance of the periciliary membrane complex that seems to play a role in regulating intracellular protein transport.	ECM/cell adhesion
NVE17236	XM_032370293	proline-rich extensin-like protein EPR1, matches brevican isoform 1 variant, partial [Homo sapiens], In zebrafish matches neurocan core protein [Danio rerio]	ECM/cell adhesion
NVE10653	XM_001625963	RAB11-binding protein RELCH homolog, Regulates intracellular cholesterol distribution from recycling endosomes to the trans-Golgi network through interactions with RAB11 and OSBP (PubMed:29514919). Functions in membrane tethering and promotes OSBP-mediated cholesterol transfer between RAB11-bound recycling endosomes and OSBP-bound Golgi-like membranes.	lipid metabolism

NVE15290	XM_032371075	uncharacterized, blast hits - stabilin-1 [Homo sapiens], Acts as a scavenger receptor for acetylated low density lipoprotein. Binds to both Gram-positive and Gram-negative bacteria and may play a role in defense against bacterial infection. When inhibited in endothelial tube formation assays, there is a marked decrease in cell-cell interactions, suggesting a role in angiogenesis.	ECM/cell adhesion
NVE18731	XM_032384894	P2Y purinoceptor 6-like, belongs to the family of G-protein coupled receptors.	lipid metabolism
NVE22894	XM_032382668	Trace amine-associated receptor 6-like, belongs to a class of G protein-coupled receptors. Trace amines are endogenous amine compounds that are chemically similar to classic biogenic amines like dopamine, norepinephrine, serotonin, and histamine. Trace amines were thought to be 'false transmitters' that displace classic biogenic amines from their storage and act on transporters in a fashion similar to the amphetamines, but the identification of brain receptors specific to trace amines indicates that they also have effects of their own. TAAR6 expressed in kidney and brain. In mouse TAAR6 is pecifically expressed in neurons of the olfactory epithelium, to discrete glomeruli predominantly localized to a confined bulb region.	neuroactive ligand-receptor
NVE16188	XR_004293154	ncRNA	ncRNA
NVE12757	XM_032372270	contactin-6 isoform X3 [Mus musculus], neuronal cell adhesion molecule isoform hh [Homo sapiens], the protein encoded by this gene is a member of the immunoglobulin superfamily. It is a glycosylphosphatidylinositol (GPI)-anchored neuronal membrane protein that functions as a cell adhesion molecule. It may play a role in the formation of axon connections in the developing nervous system.	ECM/cell adhesion
NVE5666	XM_032376690	laminin subunit beta-2, Binding to cells via a high affinity receptor, laminin is thought to mediate the attachment, migration and organization of cells into tissues during embryonic development by interacting with other extracellular matrix components. Tissue specificity -Neuromuscular synapse and kidney glomerulus.	ECM/cell adhesion
NVE5768	XM_001628429	protein HEXIM, Probable transcriptional regulator which functions as a general RNA polymerase II transcription inhibitor, Transcriptional regulator which functions as a general RNA polymerase II transcription inhibitor. In cooperation with 7SK snRNA sequesters P-TEFb in a large inactive 7SK snRNP complex preventing RNA polymerase II phosphorylation and subsequent transcriptional elongation.	TFs/transcription
NVE1877	XM_001630624	uncharacterized, blast hits - Zinc finger protein 474 (human, mouse) - ZNF494, Predicted to have metal ion binding activity and nucleic acid binding activity.	TFs/transcription

NVE13233	XM_032382318	ankyrin repeat and KH domain-containing protein 1-like, Could play pivotal roles in cell cycle and DNA regulation (PubMed:19150984). Involved in innate immune defense against virus by positively regulating the viral dsRNA receptors DDX58 and IFIH1 signaling pathways (PubMed:22328336).	immune response
NVE24958	XM_001632239	histone-lysine N-methyltransferase 2D, Histone methyltransferase. Methylates 'Lys-4' of histone H3 (H3K4me). H3K4me represents a specific tag for epigenetic transcriptional activation. Acts as a coactivator for estrogen receptor by being recruited by ESR1, thereby activating transcription.	TFs/transcription
NVE2264	XM_001630435	PLA2_2, blast hits - basic phospholipase A2 nigexine, phospholipase A2 isoform 2 precursor [Mus musculus], Phospholipase A(2) (PLA(2)) enzymes are involved in lipid metabolism and, as such, are central to several cellular processes.	lipid metabolism
NVE23108	LOC116607023	ncRNA	ncRNA
NVE12755	XM_001624903	adhesion G-protein coupled receptor D1, Orphan receptor. Signals via G(s)-alpha family of G-proteins (PubMed:22025619, PubMed:22575658). Has protumorigenic function especially in glioblastoma (PubMed:27775701), mus musculus - adhesion G protein-coupled receptor L3. Plays a role in cell-cell adhesion and neuron guidance via its interactions with FLRT2 and FLRT3 that are expressed at the surface of adjacent cells (PubMed:26235030).	ECM/cell adhesion
NVE16286	XM_032366658	AP-5 complex subunit beta-1, As part of AP-5, a probable fifth adaptor protein complex it may be involved in endosomal transport, Adaptor protein complex 1 is found at the cytoplasmic face of coated vesicles located at the Golgi complex, where it mediates both the recruitment of clathrin to the membrane and the recognition of sorting signals within the cytosolic tails of transmembrane receptors.	endocytosis/exocytosis
NVE17123	XM_001636071	pinin, Transcriptional activator binding to the E-box 1 core sequence of the E-cadherin promoter gene; the core-binding sequence is 5'CAGGTG-3'.	TF/transcription
NVE11362	XM_032362383	uncharacterized, blast hits - integrin and matrilin 1, cartilage matrix protein, isoform CRA_b [Homo sapiens], This gene encodes a member of von Willebrand factor A domain containing protein family.	ECM/cell adhesion
NVE23087	XM_032371074	volume-regulated anion channel subunit LRRC8E-like	membrane transport
NVE8748	XM_032374695	uncharacterized, collagen alpha-6(VI) chain isoform X2 [Xenopus tropicalis], von Willebrand factor type A domains, fibronectin type 2 domains, TSP type 1 repeats	ECM/cell adhesion
NVE15008	XM_032371244	early endosome antigen 1-like, EEA1 localizes exclusively to early endosomes and has an important role in endosomal trafficking.	endocytosis/exocytosis

NVE9385	XM_032365376	uncharacterized, blast hits - other uncharacterized proteins (cnidaria, moth, molluscs)	uncharacterized/cnidaria-specific
NVE5399	XM_001639086	UDP-N-acetylglucosamine--peptide N-acetylglucosaminyltransferase 110 kDa subunit, Catalyzes the transfer of a single N-acetylglucosamine from UDP-GlcNAc to a serine or threonine residue in cytoplasmic and nuclear proteins resulting in their modification with a beta-linked N-acetylglucosamine (O-GlcNAc)	protein modification
NVE6210	XM_032363854	neurogenic locus notch homolog protein 1	Notch signaling
NVE5945	XM_032376466	uncharacterized, thrombospondin 1, hemicentin-1 isoform X2 [Mus musculus], This gene encodes a large extracellular member of the immunoglobulin superfamily.	ECM/cell adhesion
NVE23760	XM_001621826	zinc metalloproteinase nas-6, Expressed in pharyngeal and body wall muscles, intestine, hypodermis and pharyngeal mc2 cells.	ECM/cell adhesion
NVE19786	XM_032384482	splicing factor 3B subunit 1, Involved in pre-mRNA splicing as a component of the splicing factor SF3B complex	RNA splicing
NVE9070	XM_032374535	uncharacterized, blast hits - chymotrypsinogen B2 precursor [Homo sapiens], Chymotrypsinis a digestive enzyme component of pancreatic juice acting in the duodenum, where it performs proteolysis, the breakdown of proteins and polypeptides.	protein degradation

Supplementary Table 3. DE genes validated with RT-qPCR, their functional classification and RNA-seq attributes.

Gene symbol	NCBI_name	NCBI_ID	Ref.genome_ID	P-adj	Log2FoldChange	Functional classification
Sorl1	sortilin-related receptor	5517308	NVE13564	0.000	-0.43007	lipid metabolism
Pla2_1	phospholipase A2 A2-actitoxin-Cgg2a	5512771	NVE23561	0.006	0.36609	lipid metabolism
Pla2_2	basic phospholipase A2 CM-III	5509975	NVE2264	0.169	-0.25764	lipid metabolism
P2ry6-like	uncharacterized (new record), P2Y purinoceptor 6-like (removed record)	116619764	NVE18731	0.187	-0.26629	lipid metabolism
Sglt5-like	sodium/glucose cotransporter 5-like	5515535	NVE18073	0.101	-0.30671	glucose metabolism
Pkm	pyruvate kinase PKM	5522266	NVE38	0.000	-0.54460	glucose metabolism
Ogt	UDP-N-acetylglucosamine--peptide N-acetylglucosaminyltransferase 110 kDa subunit	5519150	NVE5399	1.55E-01	-0.23520	post-translational modification of proteins
RPTPsigma-like	receptor-type tyrosine-protein phosphatase S-like	5514256	NVE25055	6.46E-04	-0.42214	post-translational modification of proteins
Zic4	zinc finger protein ZIC 4	5511658	NVE8653	7.01E-03	-0.37163	TF
Ets-related	uncharacterized, Ets domain	5518230	NVE9883	4.05E-04	0.43941	TF
Lrrc8c-like	volume-regulated anion channel subunit LRRC8C-like	116610263	NVE23087	1.87E-01	-0.24172	ion channel
Trpa1-like	transient receptor potential cation channel subfamily A member 1-like	5510332	NVE1653	7.09E-02	-0.29384	ion channel
Pkd1l2	uncharacterized, PKD_channel (new record); polycystic kidney disease protein 1-like 2 (old record)	5520736	NVE22455	4.91E-02	-0.32723	ion channel

Eaat1	excitatory amino acid transporter 1	5511109	NVE443	1.86E-01	-0.27145	neurotransmission
Gat2	sodium- and chloride-dependent GABA transporter 2	5503381	NVE14046	NA	NA	neurotransmission
Gat1	sodium- and chloride-dependent GABA transporter 1	5521615	NVE16531	NA	NA	neurotransmission
Cntnap2	contactin-associated protein-like 2	5510871	NVE830	6.39E-03	-0.38865	ECM/cell adhesion
Adgrf5	adhesion G protein-coupled receptor F5	116611752	NVE12757	1.16E-01	-0.26555	ECM/cell adhesion
Sdk1	protein sidekick-1	116608285	NVE20069	1.77E-01	-0.28481	ECM/cell adhesion
Adgrv1-like	adhesion G-protein coupled receptor V1-like	116601365	NVE12193	1.25E-01	-0.26955	ECM/cell adhesion
Edil3	EGF-like repeat and discoidin I-like domain-containing protein 3	116608110	NVE20550	2.39E-02	-0.35463	ECM/cell adhesion
Notch1	neurogenic locus notch homolog protein 1	5521105	NVE18894	1.69E-01	-0.27414	Notch signaling
Mpeg-1	macrophage-expressed gene 1 protein	5509442	NVE3218	1.34E-02	0.35356	immune response

Supplementary Table 4. Gene-specific siRNAs used in this study.

Designation	Target_ Reg.genome_ID	Target_NCBI_ID	Gene symbol	Sense/ antisense	Sequence	Designed by
siRNA1	NVE12024	5495825, 5504408	*NvGcm*	s	UUACCUGUGCAUUUCCUGU[dT][dT]	InvivoGen siRNA Wizard Software
				a	ACAGGAAAUGCACAGGUAA[dT][dT]	
siRNA2	NVE12024	5495825, 5504408	*NvGcm*	s	CUACCCAAUGGACUCAAGAdTdT	Sigma-Aldrich Japan
				a	UCUUGAGUCCAUUGGGUAGdTdT	
control siRNA	n/a	n/a	n/a	s	GCAACACGCAGAGTCGTAAdT	Sigma-Aldrich Japan
				a	TTACGACTCTGCGTGTTGCdT	
siRNA2	NVE443	5511109	*NvEaat1*	s	CCUAUCAAACGGCACACAUdTdT	Sigma-Aldrich Japan
				a	AUGUGUGCCGUUUGAUAGGdTdT	
siRNA3	NVE443	5511109	*NvEaat1*	s	GUAUUACUUAGCUACGACUdTdT	Sigma-Aldrich Japan
				a	AGUCGUAGCUAAGUAAUACdTdT	

Supplementary Table 5. Sequencing depths and alignment rates of the RNA-seq libraries. Libraries used for obtaining the final results are highlighted in yellow.

Sample Name	Sample ID	Total reads (millions)	Alignment rate
HTKK5DSXX_PR1396_01A8080_H1_L002_R1	siRNA1_1batch_1	79.3	76%
HTKK5DSXX_PR1396_01A8080_H1_L002_R2	siRNA1_1batch_2	79.3	
HTKK5DSXX_PR1396_02A8181_H1_L002_R1	siRNA2_1batch_1	69.7	77%
HTKK5DSXX_PR1396_02A8181_H1_L002_R2	siRNA2_1batch_2	69.7	
HTKK5DSXX_PR1396_03A8282_H1_L002_R1	control_1batch_1	56.1	77%
HTKK5DSXX_PR1396_03A8282_H1_L002_R2	control_1batch_2	56.1	
HTKK5DSXX_PR1396_04A8383_H1_L002_R1	siRNA1_2batch_1	51.8	76%
HTKK5DSXX_PR1396_04A8383_H1_L002_R2	siRNA1_2batch_2	51.8	
HTKK5DSXX_PR1396_05A8484_H1_L002_R1	siRNA2_2batch_1	76.7	78%
HTKK5DSXX_PR1396_05A8484_H1_L002_R2	siRNA2_2batch_2	76.7	
HTKK5DSXX_PR1396_06A8585_H1_L002_R1	control_2batch_1	58.8	76%
HTKK5DSXX_PR1396_06A8585_H1_L002_R2	control_2batch_2	58.8	
HTKK5DSXX_PR1396_07A8686_H1_L002_R1	siRNA1_3batch_1	54.5	76%
HTKK5DSXX_PR1396_07A8686_H1_L002_R2	siRNA1_3batch_2	54.5	
HTKK5DSXX_PR1396_08A8787_H1_L002_R1	siRNA2_3batch_1	54.3	76%
HTKK5DSXX_PR1396_08A8787_H1_L002_R2	siRNA2_3batch_2	54.3	
HTKK5DSXX_PR1396_09A8989_H1_L002_R1	control_3batch_1	70.1	75%
HTKK5DSXX_PR1396_09A8989_H1_L002_R2	control_3batch_2	70.1	
HTKK5DSXX_PR1396_10A9191_H1_L002_R1	siRNA1_4batch_1	68.1	76%
HTKK5DSXX_PR1396_10A9191_H1_L002_R2	siRNA1_4batch_2	68.1	
HTKK5DSXX_PR1396_11A9292_H1_L002_R1	siRNA2_4batch_1	64.6	76%
HTKK5DSXX_PR1396_11A9292_H1_L002_R2	siRNA2_4batch_2	64.6	
HTKK5DSXX_PR1396_12A9393_H1_L002_R1	control_4batch_1	57.6	77%
HTKK5DSXX_PR1396_12A9393_H1_L002_R2	control_4batch_2	57.6	

HTKK5DSXX_PR1396_13A9494_H1_L002_R1	siRNA1_5batch_1	50.3	77%
HTKK5DSXX_PR1396_13A9494_H1_L002_R2	siRNA1_5batch_2	50.3	
HTKK5DSXX_PR1396_14A9595_H1_L002_R1	siRNA2_5batch_1	59.1	78%
HTKK5DSXX_PR1396_14A9595_H1_L002_R2	siRNA2_5batch_2	59.1	
HTKK5DSXX_PR1396_15A9696_H1_L002_R1	control_5batch_1	55.4	77%
HTKK5DSXX_PR1396_15A9696_H1_L002_R2	control_5batch_2	55.4	
	**mean**	61.76	76%

Supplementary Table 6. GO terms and function (UniProt) of DE genes in *NvGcm* siRNA1 vs control siRNA grouped by p-adj. *DE genes identified in both siRNA2 vs cntrsiRNA and siRNA1 vs cntrsiRNA

**p-adj< 0.05, downregulated**			
**Nv_vienna_ID**	**NCBI_accession**	**Function /BLAST hit**	**GO/KEGG pathway**
NVE38*	XM_001641976	pyruvate kinase PKM	carbohydrate/energy metabolism
**p-adj < 0.05, upregulated**			
NVE9883*	XM_032362934.1	uncharactarized, ETS domain containing	TFs/transcription
NVE23912*	XM_032365681	uncharactarized	uncharacterized/cnidaria-specific
NVE83	XM_032367461	fibulin-2, this gene encodes an extracellular matrix protein, which belongs to the fibulin family.	cell adhesion
NVE13575	XM_001637235	eukaryotic translation initiation factor 3 subunit A, RNA-binding component of the eukaryotic translation initiation factor 3 (eIF-3) complex	immune response
NVE12489	XM_001637508	interferon regulatory factor 2 (IRF2) involved in cell cycle regulation	TFs/transcription
NVE8271	XM_032363372	CD59 glycoprotein [Exaiptasia pallida] involved in signal transduction for T-cell activation complexed to a protein tyrosine kinase.	cell adhesion
NVE25578	XR_004295927	ncRNA	ncRNA
NVE14422	XM_001624274	uncharacterized, domain: Rat sarcoma (Ras)-like superfamily of small guanosine triphosphatases (GTPases)	metabolism
**p-adj < 0.1, upregulated**			
NVE18185	XM_032369985	uncharacterized, similar to other uncharacterized proteins in cniderians	uncharacterized/cnidaria-specific
NVE123	XM_032369985	uncharacterized, similar to other uncharacterized proteins in cniderians	uncharacterized/cnidaria-specific

NVE2142	XM_032364650	beta-ketoacyl-ACP synthase 3 [Nocardioides sp. SLBN-35], found as a component of a number of enzymatic systems, including fatty acid synthetase (FAS)	lipid metabolism
NVE2195		uncharacterized	uncharacterized/cnidaria-specific
**p-adj < 0.2, upregulated**			
NVE8874	XM_032363200	cyclo(L-leucyl-L-leucyl) synthase-like, transferases, Acyltransferases; Aminoacyltransferases	lipid metabolism
NVE14423	XM_032371549	uncharacterized	uncharacterized/cnidaria-specific
NVE16514	XM_032366789	uncharacterized	uncharacterized/cnidaria-specific
NVE11672	XM_032372926	uncharacterized	uncharacterized/cnidaria-specific

Supplementary Table 7. Primer sequences and expected product sizes for genes used for RT-qPCR validation and cDNA contamination check.

Gene symbol	Ref.genome_ID	NCBI_ID	Primer sequence 5'-3'	Expected product size (bp)
NvNfix-like	NVE13116	5517439	Forward: GACCCTCAGTCACCATCCATC<br>Reverse: ACTGAGCTACCCTTGACCCA	cDNA: 850<br>gDNA: 1463
NvGcm	NVE12024	5495825<br>5504408	Forward:CGACCCGCGATATCAGATAAA<br>Reverse:CTGTGCATTTCCTGTGAGAGA	97
NvEaat1	NVE443	5511109	Forward: ACTCGAAGGAACCCGTTGAC<br>Reverse: GAATGGTGCATGGAAGGGTG	100
NvGat2	NVE14046	5503381	Forward: TTCTCGCCCTCTCCTCCATT<br>Reverse:GTGGTTACGCTCCTCAGACG	100
NvGat1	NVE16531	5521615	Forward: ACTCGGCAAAGAAGTCTCGG<br>Reverse: CCAGAAGGGTGAAATCGGCA	100
Nv18S	NVE20421	125568448	Forward: CTTAGATGTTCTGGGCCGCA<br>Reverse: CCAGCACGACGATGTTTCAC	100
NvGapdh	NVE23813	5512587	Forward: GGATGGACCAAGTGCCAAGAAC<br>Reverse: GCTTGCCGTTTACCTCAGGAATGA	119
NvEf1a	NVE12051	5504418	Forward: GGTTGCCTCTTCGCTTACCACT<br>Reverse: CGTTCCTGGCTTTAGGACAC	101
NvSorl1	NVE13564	5517308	Forward: TGTGACCGACCAACCATCTG<br>Reverse: GATGTGCCAGTCGTCGATCA	100
NvRPTPsigma-like	NVE25055	5514256	Forward: AACTCAGCAAACACCACCCG<br>Reverse: ACCACCCGGGAGTGATCATA	100
NvCntnap2	NVE830	5510871	Forward: CCGCCTGGATCAGTAAAGGG<br>Reverse: GTCTTGCCCTTGGTCGTGAT	100
NvZic4	NVE8653	5511658	Forward: TGCCGCGGATTGATCGTTAT<br>Reverse: AACATCGCGTTGGATGATGG	100
NvPkd1l2	NVE22455	5520736	Forward: CGGTTGATTCTCCAGAGGCT<br>Reverse: TCGTACTCGAACGGCTTTCC	100

NvTrpa1-like	NVE1653	5510332	Forward: GTAGAGGCCTTGACTCGAGC<br>Reverse: CCACCTTCCTGAAGCCACTG	100
NvSglt5-like	NVE18073	5515535	Forward: TGGCGGTATGTGGAAGTGAG<br>Reverse: ACAGCCATCATCAGACCACG	100
NvSdk1	NVE20069	116608285	Forward: CGCCTTTCTTGTCAAGTCGC<br>Reverse: CATCAGCACCGTCACTGCTA	100
NvAdgrv1-like	NVE12193	116601365	Forward: CAAGCAAGTCAACTGTTCGGA<br>Reverse: GGCAGTGTACGCAGAAAATTG	119
NvP2ry6-like	NVE18731	116619764	Forward: CAACCCTGATGTCCATGCCA<br>Reverse: GAGGGCATCGAGTGAAAGGT	100
NvAdgrf5	NVE12757	116611752	Forward: GTCAGCTGCGTTCATTGAGC<br>Reverse: ACCACGTCTAGAAACCCTGC	100
NvLrrc8c-like	NVE23087	116610263	Forward:ACTGCGCTTGGACATATAAGA<br>Reverse: TAGAGACGCGTACCGGGTTA	107
NvNotch1	NVE18894	5521105	Forward: CTGGAGTGGTAAACCCTGCC<br>Reverse: AAGATGACAAACAGGCGGTCT	100
NvEts-related	NVE9883	5518230	Forward: GTGCATTGCTGAAATTGCTTCG<br>Reverse: GTGATTGGTTGATCGGTTGCC	91
NvPla2_1	NVE23561	5512771	Forward: CCAAGAGGACGCTATGAGGC<br>Reverse: TGATCGCTTCGCCACTTCTT	96
NvEdil3	NVE20550	116608110	Forward: ATAACGAAACGGGCTGGTGC<br>Reverse: ATGCGTGAGGCTGAACAGTAA	107
NvOgt	NVE5399	5519150	Forward: CCATTGAGACCCAGCCTAGC<br>Reverse: AAGTGGTGTATTGCCAGCCA	93
NvPkm	NVE38	5522266	Forward: GCACCTTCGTTTTCGTGTCC<br>Reverse: GCGCGATGTGGATCTTCTCT	95
NvMpeg-1	NVE3218	5509442	Forward: GGTTCGGTAGTGTGAGTGGG<br>Reverse: TGTCGTAATTGCACACGGGT	100
NvPla2_2	NVE2264	5509975	Forward: GCGGGGTTAGGTTAATTGTCG<br>Reverse: TTGCAACCATTTGGCTGTCC	94
